# Contents of endogenous brassinosteroids and the response to drought and/or exogenously applied 24-*epi*brassinolide in two different maize leaves

**DOI:** 10.3389/fpls.2023.1139162

**Published:** 2023-06-02

**Authors:** Hana Marková, Danuše Tarkowská, Petr Čečetka, Marie Kočová, Olga Rothová, Dana Holá

**Affiliations:** ^1^ Department of Genetics and Microbiology, Faculty of Science, Charles University, Prague, Czechia; ^2^ Laboratory of Growth Regulators, Centre of the Region Haná for Biotechnological and Agricultural Research, Institute of Experimental Botany, Czech Academy of Sciences, v.v.i. and Palacký University, Olomouc, Czechia

**Keywords:** brassinosteroids, drought, endogenous content, exogenous application, leaf age, OJIP analysis, proline

## Abstract

Exogenously applied brassinosteroids (BRs) improve plant response to drought. However, many important aspects of this process, such as the potential differences caused by different developmental stages of analyzed organs at the beginning of drought, or by BR application before or during drought, remain still unexplored. The same applies for the response of different endogenous BRs belonging to the C_27_, C_28_-and C_29_- structural groups to drought and/or exogenous BRs. This study examines the physiological response of two different leaves (younger and older) of maize plants exposed to drought and treated with 24-*epi*brassinolide (*epi*BL), together with the contents of several C_27_, C_28_-and C_29_-BRs. Two timepoints of *epi*BL application (prior to and during drought) were utilized to ascertain how this could affect plant drought response and the contents of endogenous BRs. Marked differences in the contents of individual BRs between younger and older maize leaves were found: the younger leaves diverted their BR biosynthesis from C_28_-BRs to C_29_-BRs, probably at the very early biosynthetic steps, as the levels of C_28_-BR precursors were very low in these leaves, whereas C_29_-BR levels vere extremely high. Drought also apparently negatively affected contents of C_28_-BRs (particularly in the older leaves) and C_29_-BRs (particularly in the younger leaves) but not C_27_-BRs. The response of these two types of leaves to the combination of drought exposure and the application of exogenous *epi*BL differed in some aspects. The older leaves showed accelerated senescence under such conditions reflected in their reduced chlorophyll content and diminished efficiency of the primary photosynthetic processes. In contrast, the younger leaves of well-watered plants showed at first a reduction of proline levels in response to *epi*BL treatment, whereas in drought-stressed, *epi*BL pre-treated plants they were subsequently characterized by elevated amounts of proline. The contents of C_29_- and C_27_-BRs in plants treated with exogenous *epi*BL depended on the length of time between this treatment and the BR analysis regardless of plant water supply; they were more pronounced in plants subjected to the later *epi*BL treatment. The application of *epi*BL before or during drought did not result in any differences of plant response to this stressor.

## 1 Introduction

Drought is one of the most significant stressors affecting agricultural production worldwide; thus, it is crucial to alleviate its negative effect on plants. Various approaches to achieve this are possible. Aside from classical breeding and genetic engineering, the treatment of plants with chemical compounds such as antioxidants, phytohormones, polyethylene glycol, *etc.*, has also been reportedly tried. One group of these compounds are brassinosteroids (BRs): steroid phytohormones showing a wide range of functions in the regulation of plant growth and development and significantly participating in plant defense against diverse environmental stressors (Ahammed et al., 2022).

More than 170 studies focused on BRs and drought stress have been published during the past three decades. Most of these studies were performed with plants treated with exogenous BRs, although mutants in genes associated with BR synthesis or signaling have also been utilized (for review see, *e.g.*, Holá, 2019; Sidhu and Bali, 2022). However, the results of these studies are so variable that it is still impossible to draw a clear conclusion about the role of BRs in plant response to drought. To demonstrate this, let us randomly choose just two parameters strongly associated with plant drought response: *i)* catalase activity (CAT; an important enzymatic antioxidant) and *ii)* the proline content (important osmoprotectant as well as a non-enzymatic antioxidant). Chandrasekaran et al. (2017) and Sivakumar et al. (2017) reported a reduced CAT activity in drought-stressed BR-treated tomato plants compared to non-treated ones. Completely opposite results were published in some previous analyses performed with the same species (Behnamnia et al., 2009; Yuan et al., 2010). Similarly, in several studies made with various drought-stressed plant species, reduced levels of proline were observed after BR treatment (Li et al., 2012; Gursude et al., 2014; Ahmed et al., 2017; Hemmati et al., 2018). Other studies on this topic, where proline was also analyzed, reported its increased levels in drought-stressed BR-treated plants (*e.g.*, Zhu et al., 2014; Shahana et al., 2015; Younesian et al., 2017; Lv et al., 2020).

The above-mentioned variability of the data on the relationship between BRs and plant drought response results from many factors such as the inter- or intra-species differences, the developmental stage of analyzed plants/organs, duration of the drought period, the method utilized for the drought simulation, the BR type and concentration, the method of plant treatment with BRs, timepoints of BR application, *etc*. (for more details see Holá, 2019). Unfortunately, studies that would purposely focus on the effects these possible sources of variability can have on plant responses to BRs are very rare. Thus, we decided to focus on two following aspects in order to ascertain how they could affect plant responses to BRs: *i)* to analyze the response of two different leaves (already developed and still developing ones at the beginning of drought period) of plants exposed to drought and treated with BRs, and *ii)* to compare two timepoints of BR application (prior to drought and during drought).

There is extremely little knowledge on the possible different response of leaves already developed before drought compared to leaves developing during this abiotic stress to the treatment with exogenous BRs. So far, only Gomes et al. (2013) examined some photosynthetic parameters in three different leaves of papaya stressed by drought and treated with BRs. They reported that BR application led to slightly more pronounced drought-induced degradation of chlorophyll (Chl) in the older leaves compared to the younger ones. On the other hand, Wang et al. (2015) compared the stomatal density, width and length in BR-treated drought-stressed young and mature leaves of grapevine plants and found that BR treatment significantly affected these parameters only in the young leaves. This seems to indicate that fully developed leaves could indeed respond to the combination of drought and BR treatment in a different manner than leaves that are still developing. However, these differences could depend on both plant species and/or the respective evaluated parameter.

Similarly, there is very little knowledge on the potentially different impact of BR treatment before or during drought. If we compare the results of studies dealing with BR application only before the stress period with studies where BRs were applied only during the stress period, we can perhaps discern slightly different responses of plants to these treatments. For example, Behnamnia et al. (2009) sprayed tomato plants before the onset of drought and found mostly increased activities of several antioxidant enzymes including CAT in their drought-stressed BR-treated plants. On the other hand, Sivakumar et al. (2017) treated plants of the same species with BRs during stress and in this case, the CAT activity decreased. However, due to the high variability of many different aspects of experimental setups in BR/drought studies, it is very complicated to ascertain whether there truly could be some significant differences between these two main potential timepoints of BR application. So far, only two groups of authors applied BRs at these timepoints in the same study in order to purposely compare the respective effects. Unfortunately, in both cases, a different type of BR application for each timepoint was used. Hashemi et al. (2015) compared the effect of seed priming before drought and leaf spraying during the stress period. They found almost no difference between these two approaches/timepoints, but each method required a different BR concentration for the maximum effect. In an earlier experiment by Farooq et al. (2009), no difference between the effect of seed soaking prior to drought and leaf spraying during the drought period was observed.

In addition to the two above-mentioned aims of our study, we further wanted to examine the effect of drought per se and in combination with the exogenous application of BRs on their endogenous levels in leaves (and roots). Our focus was again on the possible differences caused by the leaf age/development before or during the drought period. Several studies revealed that plant exposure to drought can cause changes in BR levels in pea (Jager et al., 2008), soybean (Janeczko et al., 2011), rice (Ding et al., 2014; Zhang et al., 2020; Zhang et al., 2022b), barley (Gruszka et al., 2016; Malaga et al., 2020), tobacco (Duan et al., 2017), foxtail millet (Tang et al., 2017), Chinese cabbage, white cabbage and kale (Pavlović et al., 2018), maize (Tůmová et al., 2018), Kentucky bluegrass (Chen et al., 2019) and tomato (Nie et al., 2019). However, different plant species showed a rather varied response in this respect and the majority of these studies analyzed either only the contents of total BRs or the contents of the two most biologically active BRs, *i.e.*, castasterone (CS) and/or brassinolide (BL). No other member of big BR family was studied. Regarding the effects of the exogenous BR application on the endogenous BR levels, all studies dealing with the such topic have been made under non-stress conditions (with the exception of Efimova et al., 2014, who studied salinity stress) and focused again mostly on the total BR content (Mao et al., 2017; Fu et al., 2019; Nie et al., 2019; Setsungnern et al., 2019; Chen et al., 2021; Zhang et al., 2021; Liu et al., 2022). Beside CS and BL, some authors also determined the contents of some C_27_- and C_29_-BRs (Janeczko and Swaczynová, 2010; Janeczko et al., 2010; Janeczko et al., 2011; Filek et al., 2019; Tarkowská et al., 2020) and more detailed analyses including some BR biosynthetic precursors were performed by Bajguz et al. (2019) and Chmur and Bajguz (2021). However, all these studies were made with plants under non-stress conditions. To our knowledge, no data on the possible changes in the contents of various individual endogenous BRs or their precursors, induced by the treatment of plants with exogenously applied BRs under drought conditions, are available at this time.

Thus, the purpose of our study can be summarized into several main objectives. Firstly, we wanted to examine potential differences between the response of younger and older leaves to exogenous BR application, both in drought conditions and conditions of sufficient water supply (we hypothesized that the older leaves, as well as the leaves of stressed plants, should probably show a more pronounced response, because BRs generally seem to function particularly under suboptimal conditions). The second objective was comparison of the effect of BR application before or during drought period (our hypothesis here was that the application during drought could affect plants more strongly, because they were already stressed and BRs could thus immediately show their anti-stress effects). The third objective consisted in the evaluation of the general effect that plant treatment with 24-*epi*BL has on the portfolio/contents of various BRs belonging to three different structural groups, and the potential differences in this respects caused by the length of time between exogenous BR application and endogenous BR determination (we hypothesized that the effects of exogenous BRs will probably diminish with time and that plants treated with 24-*epi*BL, a representative of C_28_-BRs, could probably divert their BR biosynthesis into C_29_- or C_27_-BR biosynthetic pathways). Finally, we also wanted to examine whether the contents of individual BRs differ between younger and older leaves (and roots) and whether these contents change due to drought treatment (we expected a positive answer but could not predict the nature of these differences and/or changes).

## 2 Materials and methods

### 2.1 Plant material and cultivation conditions

The drought-sensitive maize (*Zea mays* L.) inbred line 2023 (Benešová et al., 2012) from the CEZEA Maize Breeding Station (*Čejč, Czech Republic*) was used for the experiments. Plants were grown in plastic pots (15 cm diameter, 23 cm height; 1 plant per pot) filled with the mix (10:1) of garden soil (Garden Compost, *Agro CS*, *Czech Republic*) and sand (Spielsand Sahara sand, *WECO, Germany*) at the greenhouse facility of the Faculty of Science, Charles Univerzity, Prague, the Czech Republic, 50°04’ N, 14°25’ E, 238 m above the sea level) under semi-controlled conditions during the spring season (April, May). The conditions in the greenhouse were: the average temperature of 25/20°C, the average relative air humidity of 60/80% day/night, natural irradiance, and watering of plants with tap water as necessary. Moderate drought stress was simulated by the cessation of watering starting at the day 35 after the date of sowing and maintained for two weeks.

The first measurements/samplings (Timepoint 1) were made from the 32 d-old plants (at this time all plants had three fully developed leaves). The second measurement/sampling point (Timepoint 2) was executed after additional 3 d when the drought simulation started. Timepoint 3 occurred after 7 d of drought and the last samplings/measurements (Timepoint 4) were at the end of the drought period. All morphological, physiological, and biochemical parameters were assessed during these four timepoints (with the exception of the determination of endogenous BR contents, which was done only at Timepoint 4). The volumetric soil water content was 24.1% at Timepoint 1, 22.6% at Timepoint 2, 22.5% for the normally-watered plants and 13.8% for stressed plants at Timepoint 3, and 21.8% for normally-watered plants and 9.3% for stressed plants at Timepoint 4.

Separate plants were used for *i)* plant morphology assessment (16 biologic replicates), *ii)* determination of the relative water content (RWC), photosynthetic pigments contents and Chl fluorescence measurements (8 biologic replicates); *iii)* the membrane damage index (MDI) (6 biologic replicates); *iv)* gas exchange measurements (6 biologic replicates); *v)* determination of the malondialdehyde (MDA) content (8-9 biologic replicates); *vi)* determination of the proline content (9 biologic replicates); and *vii)* determination of the BR contents (3-4 biologic replicates). In all cases, the 3^rd^ (*i.e.*, fully developed at the beginning of drought) or the 4^th^ (*i.e.*, developing during drought) leaves were used for the physiological/biochemical analyses. In addition, the BR contents were also determined in the roots.

### 2.2 BR treatments

The 10^-6^ M aqueous solution of 24-*epi*brassinolide (*epi*BL; (22*R*,23*R*,24*R*)-2α,3α,22,23-tetrahydroxy-24-methyl-7-oxa-7-homo-5α-cholestan-6-one; *Sigma-Aldrich-Merck*) used for treatments was prepared from the 10^-4^ M stock solution containing distilled water:96% ethanol (10:1; ethanol was used for the dissolution of *epi*BL and water was then gradually added with continuous stirring till the stock solution of fully dissolved *epi*BL was achieved) and contained also 0.05% of nonionic detergent Tween^®^ 20. The corresponding control solution (C) had the same composition except *epi*BL. The concentration of *epi*BL was chosen based on our previous experiments (unpublished data). One group of plants was sprayed with the *epi*BL solution at Timepoint 1 (the BR1 variants, application prior to drought), another group of plants was sprayed at Timepoint 3 (the BR2 variants, application during drought). The respective samplings/measurements at these timepoints were always done before the *epi*BL treatment. In all cases, the whole aboveground part of plants was always sprayed (the amount of solution applied per plant was approximately 10 ml). Thus, at the end of the experiments (Timepoint 4), six experimental variants were available: three originated from plants that were well-watered during the whole experiment, and another three were from plants exposed to drought simulation.

### 2.3 Plant development and morphology

Plant development was monitored by counting the number of fully developed leaves in all plants of the respective variants throughout the whole experiment. The dry masses of the shoot (DMS) and roots (DMR) together with the plant height (measured from the surface of the soil in the pots to the youngest fully developed leaf node) were also assessed.

### 2.4 RWC and gas exchange

The RWC was evaluated by a standard method described in Čatský (1960). A small piece (approx. 3-4 cm^2^) was cut from the respective leaf and immediately weighed (FW). Then it was put into distilled water and left saturating for 5 hours. At that moment the saturated weight (SW) was obtained and the leaf piece was left to dry completely at 80°C and again weighed (DW). The RWC was calculated as (FW-DW)/(SW-DW).

The net transpiration rate (E), the stomatal conductance (g_S_) and the net photosynthetic rate (P_N_) were determined by gasometric measurements using the portable LCpro+ device (*ADC BioScientific, Hoddesdon, UK*) with the following conditions in the measuring chamber: the temperature 25°C, the ambient CO_2_ concentration 550 ± 50 μL L^-1^, the airflow rate 205 ± 30 μmol s^-1^, irradiance 650 μmol m^-2^ s^-1^ of photosynthetically active radiation. These measurements were performed between 9:00 and 12:00 AM, Central European Time, at the middle part of the respective leaves.

### 2.5 Photosynthetic pigments contents and Chl fluorescence (OJIP) analysis

Six small discs (0.6 cm^2^) were cut from the middle part of the leaf blade and incubated for 7 days in 5 mL of *N,N*-dimethylformamide at 4°C in the dark (the extracts were stirred several times during this period). After extraction, the absorbances at 480, 664, 647 and 710 nm were measured spectrophotometrically. The contents of Chl *a*, *b* and total carotenoids (Car) were evaluated using the formulae of Wellburn (1994).

Chl fluorescence was measured on the top side of dark-adapted (20 min) leaves with the portable fluorometer FluorPen FP100max (*Photon System Instruments, Brno, Czech Republic*) between 8:30 and 9:00 AM, Central European Time. The measurements were started with a saturating pulse (blue light, 455 nm, 3000 μmol m^-2^ s^-1^). After that the Chl fluorescence transient was recorded at a time scale from 10 µs to 2 ms, representing the so-called OJIP curve. Fluorescence values F_0_ (the initial fluorescence intensity recorded at 40 μs), F_K_ (the fluorescence intensity at the K-step of the OJIP curve, 300 μs), F_J_ (the fluorescence intensity at the J-step of the OJIP curve, 2 ms), F_I_ (the fluorescence intensity at the I-step of the OJIP curve, 30 ms), and F_M_=F_P_ (the maximum fluorescence intensity) were used for the calculations of various parameters of the JIP test according to Strasser et al. (2000) and Stirbet and Govindjee (2011). These parameters can be utilized for the description of the performance of various steps of the photosynthetic electron transport chain (PETC; see **Supplementary Table 1** for their definitions and formulae).

To obtain further information on the primary photosynthetic processes in various *epi*BL-treated/control or stressed/well-watered experimental variants, the normalizations of chlorophyll fluorescence transients leading to calculations of relative variable fluorescences W_OI_, W_OJ_ and W_OK_ were performed according to Yusuf et al. (2010). The positions/amplitudes of the W_OI_ curves after the I-step can inform about the size of the pool of end electron acceptors in the PETC after Photosystem (PS) I (the lower positions reflect the lower size of this pool). W_OJ_ and W_OK_ serve for further calculations of so-called difference kinetics (ΔW), which are always based on comparisons of some treatment *versus* the respective control. In our case they were based either on the comparison of *epi*BL-treated (BR1, BR2) and control (C) plants subjected to the same watering conditions, or on the comparison of non-watered and well-watered plants subjected to the same type of treatment. The difference kinetics ΔW_OJ_ and ΔW_OK_ enabled us to visualize K- and L-bands of OJIP curves, respectively. If the respective K-band showed a negative amplitude, the state of the oxygen-evolving complex (OEC) of PSII was more active in the BR1/BR2 variants than in C plants (or, alternatively, in the non-watered plants than in the well-watered ones), if it showed a positive amplitude, the reverse was true. The position/amplitude of the L-band which yields information on the energetic connectivity among individual PSII units in the PETC of compared variants, can be interpreted in a similar way (Yusuf et al., 2010).

### 2.6 Membrane damage and proline content

The MDI was determined according to Sullivan (1972). Twelve small discs (0.6 cm^2^) were cut from the middle part of the leaf blade and incubated in distilled water for 24 h at 4°C. After adjustment to the room temperature, the electrical conductances of the samples were measured using the Gryf 158 conductometer (*Gryf HB spol. s.r.o., Czech Republic*). The samples were then boiled in a water bath for 15 min, again adjusted to room temperature and the conductances were measured again. The MDI was calculated as the ratio of the conductance values before and after boiling.

To determine the MDA content, a modified method by Hodges et al. (1999) was utilized. 0.2 g of leaf tissue were ground in liquid nitrogen and then homogenized in 80% ethanol and centrifuged at 14000× *g* and 4°C for 20 min. The supernatant was added separately to the thiobarbituric acid (TBE) reaction mixture (*TBA+*; 0.65% TBA, 20% trichloroacetic acid and 0.01% butylated hydroxytoluene) and the *TBA-* reaction mixture (20% trichloroacetic acid and 0.01% butylated hydroxytoluene). In the next step, the samples with *TBA+/-* reaction mixtures were temperated at 95°C for 30 min. After cooling to room temperature, the samples were centrifuged again (14000× *g*, 4°C, 20 min). The absorbance of the supernatant was measured at 440 nm, 532 nm and 600 nm. The MDA content was calculated according to Hodges et al. (1999).

The proline content was determined spectrophotometrically according to Bates et al. (1973). The frozen samples were homogenized in 3% aqueous sulfosalicylic acid and treated with acid-ninhydrin and acetic acid. This reaction mixture was boiled in a water bath for 30 min and then cooled on ice. 3 mL of toluene were added to the cooled samples. After phase stabilization (approximately 20 min at room temperature), the absorbance of the toluene phase was measured at 520 nm and the proline content was calculated based on its calibration curve.

### 2.7 BR contents

Contents of various BRs were determined after their extraction and purification using ultra-high performance liquid chromatography (UHPLC) followed by mass spectrometry ((+)ESI-MS/MS) analysis according to Tarkowská et al. (2016).

Frozen maize tissue samples of 50 mg FW were homogenized to a fine consistency using 2.8-mm zirconium oxide beads (*Retsch GmbH & Co. KG, Haan, Germany*) and a MM 400 vibration mill (*Retsch GmbH & Co. KG, Haan, Germany*) at a frequency of 27 Hz for 3 min. The samples were then extracted overnight with stirring at 4°C using a benchtop laboratory rotator Stuart SB3 (*Bibby Scientific Ltd., Staffordshire, UK*) after adding 1 mL ice-cold 60% acetonitrile and a mixture of stable isotope internal standards (*OlChemIm Ltd., Olomouc, Czech Republic*) including [^2^H_3_]BL, [^2^H_3_]CS, [^2^H_3_]24-*epi*BL, [^2^H_3_]24-*epi*CS, [^2^H_3_]28-norBL, [^2^H_3_]28-norCS, [^2^H_3_]TY, [^2^H_3_]campestanol, [^2^H_3_]campesterol, [^2^H_3_]6-deoxocathasterone, [^2^H_3_]6-deoxotyphasterol and [^2^H_3_]6-oxocampestanol. The samples were further centrifuged, purified on polyamide SPE columns (*Supelco, Bellefonte, PA, USA*) and then analyzed by UHPLC-MS/MS (*Micromass, Manchester, UK*). The data were analyzed using Masslynx 4.2 software (*Waters, Milford, MA, USA*) and the BR contents were finally quantified by the standard isotope-dilution method (Rittenberg and Foster, 1940).

### 2.8 Statistical analysis

The original data are shown in **Supplementary File 1**. Mean values and standard deviations (SD) were calculated for all parameters. The data were first subjected to Welch´s ANOVA. Where appropriate, the pairwise differences between experimental variants were analyzed using Welch’s *t*-tests, for multiple comparisons, Games-Howell *post hoc* tests were applied. The data from the 3^rd^ and 4^th^ leaves were statistically analyzed separately because it was not technically possible to determine BR contents (or some of the evaluated biochemical parameters) in all sample variants (3^rd^ and 4^th^ leaves, roots) together in one run and we did not want to introduce artificial differences to our data that could be actually caused by the different analytical runs.

## 3 Results

### 3.1 Timepoint 1

The data from Timepoint 1 are shown in **Supplementary File 1**; they were obtained only in order to characterize basal levels of plant performance prior to any *epi*BL treatment or stress induction. Thus, there is no point in their presentation here or in the subsequent discussion.

### 3.2 Timepoint 2 (no drought, early epiBL treatment)

**Figure 1:**
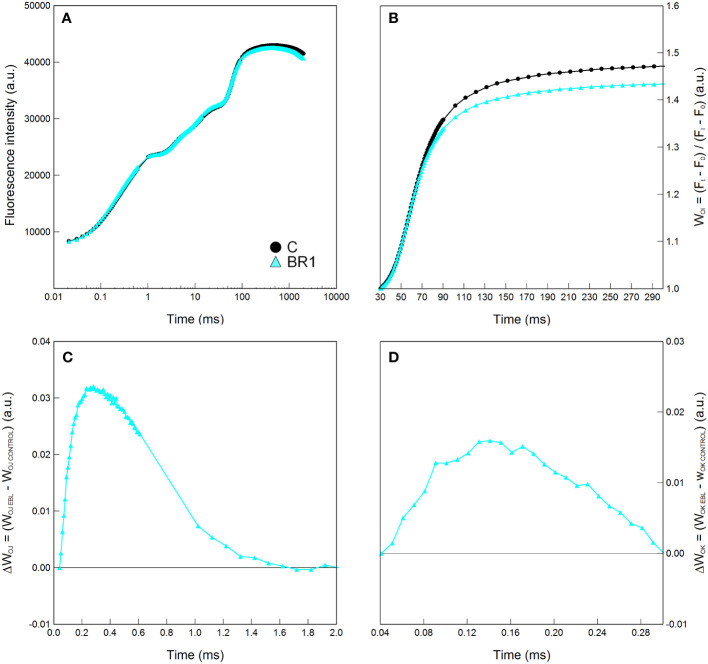
Graphical analysis of the OJIP chlorophyll fluorescence data measured in maize 3^rd^ leaves at Timepoint 2. Plants were well-watered and either treated with 24-*epi*brassinolide (the BR1 variant) or with water (the C variant). The OJIP curve **(A)**, the relative variable fluorescence W_OI_ **(B)** and the difference kinetics ΔW_OJ_ **(C)** and ΔW_OK_ **(D)** are shown. Only the part between the I and P points of the OJIP curve is shown for the W_OI_. ΔW_OJ_ reveals the K-band; ΔW_OK_ reveals the L-band and were calculated from the comparison of BR1 and C variants; the latter are represented by the zero point of the respective y axes in graphs in panels **(C**, **D)**. Mean values (n = 8) are shown. a.u., alternative units.

The BR1 treatment significantly reduced levels of proline in the 4^th^ leaves whereas in the 3^rd^ leaves this treatment had no effect (**Supplementary Table 2**). Slight OEC inactivation and reduction of the energetic connectivity among PSII complexes (inferred from the positions of the ΔW_OJ_ or ΔW_OK_ curves above zero), as well as moderate reduction in the pool size of end electron acceptors in the PETC (inferred from the relative positions of the respective W_OI_ curves) in the 3^rd^ leaves of the *epi*BL-treated plants (but not in the 4^th^ leaves), was suggested by the graphical analysis of Chl fluorescence curves (**Figure 1**, **Supplementary Figure 1**). Aside from this, plants of the BR1 and C variants did not significantly differ in any other parameter at this timepoint (**Supplementary Table 2**).

### 3.3 Timepoint 3 (drought, early epiBL treatment)

**Figure 2:**
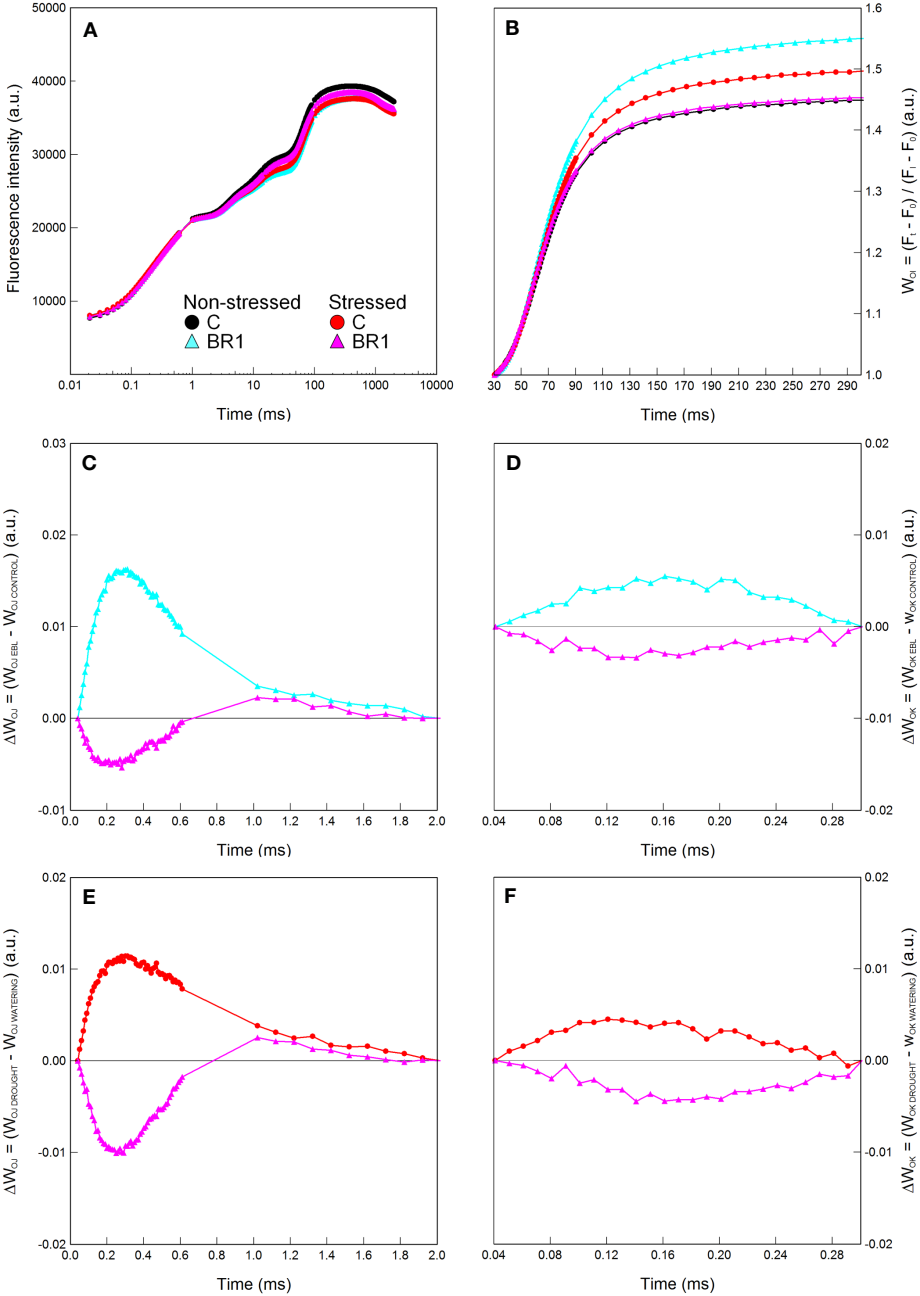
Graphical analysis of the OJIP chlorophyll fluorescence data measured in maize 4^th^ leaves at Timepoint 3. Plants were either treated with 24-*epi*brassinolide (the BR1 variant) or with water (the C variant) and subjected to normal watering (non-stressed plants) or 7 d of withholding water (stressed plants). The OJIP curve **(A)**, the relative variable fluorescence W_OI_ **(B)** and the difference kinetics ΔW_OJ_ **(C, E)** and ΔW_OK_ **(D, F)** are shown. ΔW_OJ_ and ΔW_OK_ were calculated either from the comparison of the respective BR1 and C variants subjected to the same watering regime **(C, D)** or from the comparison of the respective non-stressed and stressed plants subjected to the same type of treatment **(E, F)**. For other information see legend to **Figure 1**.

One week of drought stress significantly reduced the plant height in comparison to the well-watered plants, as well as the DMS of the *epi*BL-treated plants (**Table 1**). RWC, E, P_N_ and g_S_ values also significantly decreased due to drought (**Table 1**; for RWC, this decrease was statistically significant only in the 3^rd^ leaves of plants treated with the control solution, for the gas exchange parameters, it was significant only in the 4^th^ leaves of both *epi*BL-treated and control plants and in the 3^rd^ leaves of the *epi*BL-treated plants). Similar drought-induced reduction was observed for the contents of photosynthetic pigments in the 3^rd^ leaves; this was more pronounced in the control plants compared to the *epi*BL-treated ones (**Table 1**). One week of drought led also to an increase of the MDI values in the leaves of the control plants and the proline content in the leaves of both control and *epi*BL-treated plants, but not to any significant changes in the MDA content (**Table 1**). The efficiency of the PETC did not seem to be particularly affected by drought at this timepoint (**Figure 2**, **Supplementary Figure 2**); the exceptions being mostly the parameters related to the electron transport after PSII (φ_RE01_, ψ_RE01_, δ_RE01_, RE_01_/RC, PI_TOTAL_) in the 4^th^ leaves of the *epi*BL-treated plants (**Table 1**).

**Table 1:** Selected morphological, physiological and biochemical parameters measured at Timepoint 3 in maize leaves.

	3^rd^ leaf (or the whole plant *)				4^th^ leaf			
	Well-watered plants		Drought-stressed plants		Well-watered plants		Drought-stressed plants	
	C	BR1	C	BR1	C	BR1	C	BR1
Plant height (mm) *	*190.86 ± 11.16*	*205.88 ± 29.79*	*177.20 ± 14.62*	*166.63 ± 21.45*				
Number of leaves *	4.00 ± 0.00	4.00 ± 0.00	4.00 ± 0.00	4.00 ± 0.00				
DMS (g) *	2.05 ± 0.30	*2.56 ± 0.64*	1.87 ± 0.50	*2.00 ± 0.46*				
DMR (g) *	0.66 ± 0.15	0.70 ± 0.15	0.63 ± 0.10	0.64 ± 0.15				
RWC (%)	*98.42 ± 0.79*	98.39 ± 1.08	*96.47 ± 1.41*	97.37 ± 0.95	97.84 ± 0.62	97.19 ± 1.26	92.53 ± 10.79	94.22 ± 3.06
E (mmol H_2_O m^-2^ s^-1^)	2.09 ± 0.77	1.87 ± 0.43	1.57 ± 0.21	1.53 ± 0.36	*2.02 ± 0.52*	*2.00 ± 0.71*	*1.33 ± 0.17*	*1.17 ± 0.60*
g_S_ (mol m^-2^ s^-1^)	0.13 ± 0.02	*0.14 ± 0.02*	0.10 ± 0.04	*0.09 ± 0.03*	*0.10 ± 0.03*	*0.10 ± 0.03*	*0.05 ± 0.02*	*0.05 ± 0.03*
P_N_ (μmol CO_2_ m^-2^ s^-1^)	21.29 ± 1.23	*20.66 ± 1.37*	16.85 ± 4.85	*16.04 ± 4.25*	*20.20 ± 3.26*	*21.69 ± 2.39*	*12.29 ± 5.37*	*12.37 ± 7.13*
Chl *a* content (g kg^-1^)	*17.86 ± 0.97*	*17.71 ± 0.90*	*15.97 ± 1.08*	*16.03 ± 0.72*	15.89 ± 0.76	15.26 ± 1.28	15.16 ± 1.14	14.54 ± 1.14
Chl *b* content (g kg^-1^)	*4.91 ± 0.26*	4.77 ± 0.49	*4.41 ± 0.33*	4.12 ± 1.05	4.57 ± 0.30	4.34 ± 0.31	4.27 ± 0.34	4.10 ± 0.14
Car content (g kg^-1^)	*3.29 ± 0.20*	3.12 ± 0.21	*2.92 ± 0.14*	2.93 ± 0.15	2.78 ± 0.13	2.75 ± 0.10	2.68 ± 0.18	2.63 ± 0.18
MDI (%)	30.46 ± 2.60	30.76 ± 1.82	28.70 ± 1.95	29.12 ± 0.83	*31.21 ± 2.08*	31.45 ± 1.06	*34.31 ± 2.14*	32.46 ± 1.66
MDA content (nmol g^-1^)	19.59 ± 12.79	12.38 ± 10.35	16.84 ± 13.95	13.40 ± 5.37	23.34 ± 6.22	30.82 ± 11.42	32.32 ± 17.46	26.69 ± 7.25
Proline content (mg g^-1^)	*25.59 ± 4.10*	*26.46 ± 4.46*	*58.57 ± 33.12*	*49.81 ± 16.21*	*58.81 ± 12.88*	*47.25 ± 9.49*	*448.30 ± 129.32*	*547.49 ± 191.93*
φ_P0_	0.78 ± 0.02	0.78 ± 0.03	0.77 ± 0.02	0.77 ± 0.01	*0.79 ± 0.01*	0.77 ± 0.04	*0.77 ± 0.02*	0.78 ± 0.01
φ_E0_	1.41 ± 0.03	1.41 ± 0.05	1.40 ± 0.06	1.41 ± 0.01	1.39 ± 0.03	1.38 ± 0.04	1.38 ± 0.03	1.38 ± 0.03
φ_RE01_	0.22 ± 0.02	0.22 ± 0.03	0.21 ± 0.03	0.21 ± 0.02	0.25 ± 0.01	*0.27 ± 0.03*	0.25 ± 0.03	*0.24 ± 0.02*
φ_D0_	0.22 ± 0.02	0.22 ± 0.02	0.23 ± 0.02	0.23 ± 0.01	*0.21 ± 0.01*	0.23 ± 0.04	*0.23 ± 0.02*	0.22 ± 0.01
ψ_E0_	0.55 ± 0.01	0.55 ± 0.01	0.55 ± 0.01	0.55 ± 0.01	0.57 ± 0.01	0.55 ± 0.03	0.56 ± 0.02	0.56 ± 0.01
ψ_RE01_	0.29 ± 0.02	0.28 ± 0.04	0.27 ± 0.04	0.27 ± 0.02	0.31 ± 0.01	*0.36 ± 0.05*	0.33 ± 0.04	*0.31 ± 0.02*
δ_RE01_	0.52 ± 0.04	0.50 ± 0.07	0.49 ± 0.07	0.49 ± 0.04	0.55 ± 0.02	*0.64 ± 0.11*	0.60 ± 0.09	*0.56 ± 0.04*
γRC2	0.56 ± 0.01	0.56 ± 0.02	0.56 ± 0.01	0.56 ± 0.01	0.58 ± 0.01	0.57 ± 0.02	0.57 ± 0.02	0.58 ± 0.01
ABS/RC	0.80 ± 0.04	0.77 ± 0.05	0.79 ± 0.03	0.79 ± 0.04	0.72 ± 0.03	0.76 ± 0.08	0.75 ± 0.05	0.74 ± 0.02
TP_0_/RC	0.61 ± 0.03	0.60 ± 0.02	0.61 ± 0.01	0.61 ± 0.03	0.57 ± 0.02	0.58 ± 0.03	0.58 ± 0.03	0.57 ± 0.01
ET_0_/RC	0.34 ± 0.01	0.33 ± 0.01	0.34 ± 0.01	0.34 ± 0.01	0.32 ± 0.02	0.32 ± 0.01	0.32 ± 0.01	0.32 ± 0.01
RE_01_/RC	2.16 ± 0.23	2.20 ± 0.37	2.29 ± 0.41	2.28 ± 0.25	1.82 ± 0.09	*1.65 ± 0.17*	1.76 ± 0.19	*1.84 ± 0.14*
DI_0_/RC	0.18 ± 0.02	0.17 ± 0.03	0.18 ± 0.01	0.18 ± 0.02	0.16 ± 0.01	0.18 ± 0.05	0.18 ± 0.03	0.16 ± 0.01
PI_ABS_	5.52 ± 0.88	5.78 ± 1.32	5.39 ± 0.59	5.06 ± 0.52	6.60 ± 0.66	5.73 ± 1.96	5.62 ± 1.33	6.17 ± 0.68
PI_TOTAL_	6.31 ± 1.37	5.97 ± 1.95	5.27 ± 1.55	4.77 ± 0.79	8.24 ± 1.20	*10.41 ± 2.25*	8.60 ± 2.57	*7.80 ± 1.45*

Plants were either treated with 24-epibrassinolide (the BR1 variant) or with water (the C variant) and subjected to normal watering (well-watered plants) or 7 d of withholding water (drought-stressed plants). Statistically significant differences (p ≤ 0.05) according to Welch´s t-tests between the respective C and BR1 variants subjected to the same cultivation conditions are shown in bold, statistically significant differences between the respective well-watered and drought-stressed variants treated with the same solution are shown in italics. For the explanation of abbreviations see the Material and Methods section of the article, for the biological meaning of the JIP test parameters see **Supplementary Table 1**.

The early *epi*BL treatment had no statistically significant effect on the values of most parameters evaluated in plants subjected to either well-watered or drought conditions, with the exception of the DMS which was greater in the well-watered BR1 plants compared to the respective C variant. Additionally, their 4^th^ leaves were again characterized by significantly lower contents of proline. They also showed higher values of the JIP test parameters related to the electron transport after PSII (**Table 1**) and had a greater size of the pool of end electron acceptors in the PETC (**Figure 2**); however, this did not apply to the 3^rd^ leaves of these plants or to the drought-stressed *epi*BL-treated plants (**Figure 2**, **Supplementary Figure 2**). The inactivation of the OEC in the 4^th^ leaves of the *epi*BL-treated well-watered plants appeared at this timepoint as well, although to a less extent than in the 3^rd^ leaves at the Timepoint 2. However, in the drought-stressed plants, the *epi*BL treatment positively affected the OEC function (**Figure 2**). No particular effect of the early *epi*BL treatment on the energetic connectivity between PS II units was found at this timepoint (**Figure 2**).

### 3.4 Timepoint 4 (drought, early and late epiBL treatments)

**Figure 3:**
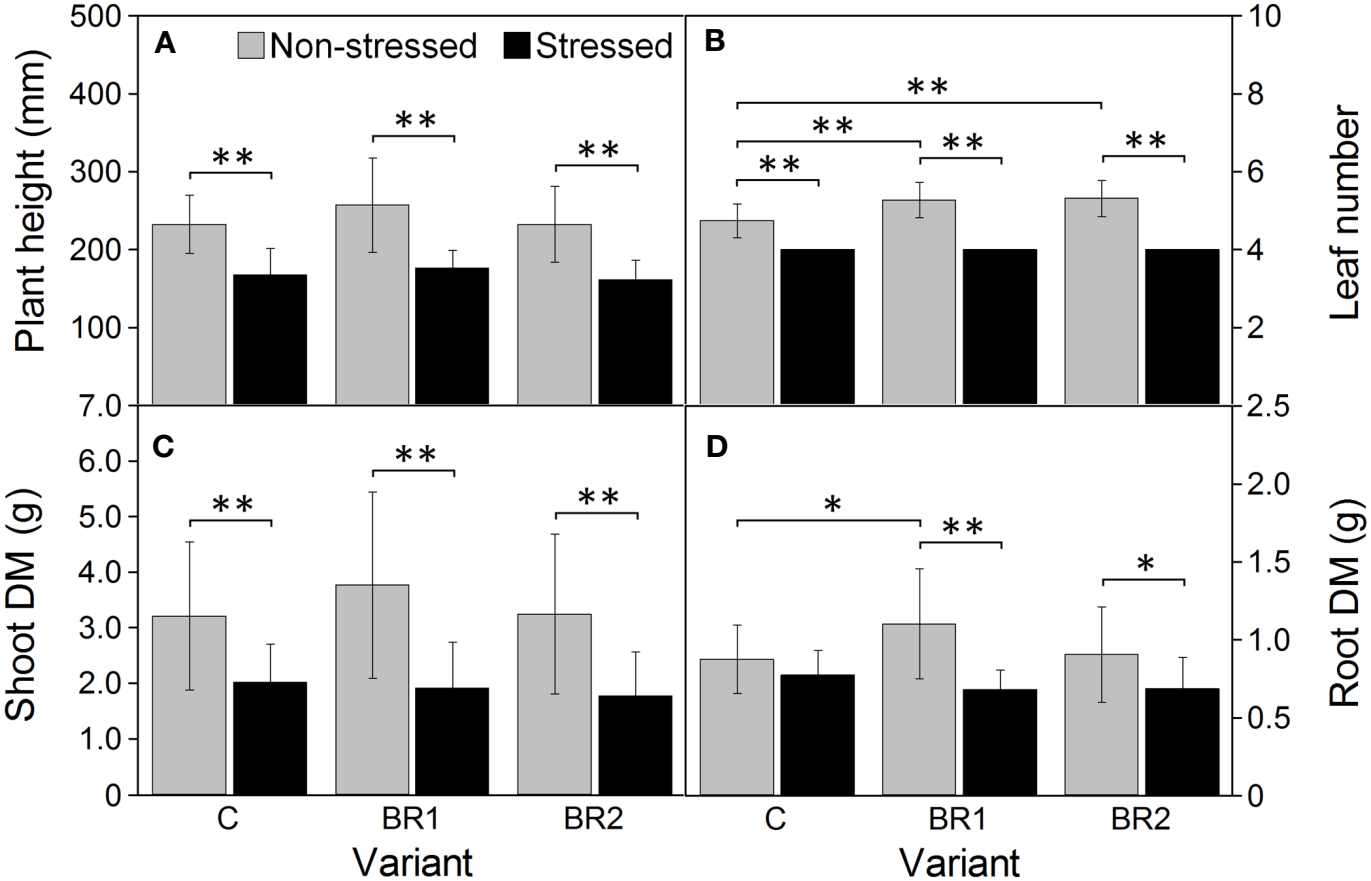
Plant height **(A)**, the number of fully developed leaves **(B)**, the dry mass of shoot **(C)** and the dry mass of roots **(D)** measured in maize at Timepoint 4. Plants were subjected to normal watering (non-stressed plants) or 14 d of withholding water (stressed plants) and treated with 24-*epi*brassinolide (the BR1 and BR2 variants) or with water (the C variant) either before the start of drought period (C, BR1) or during the drought period (BR2). Mean values ± SD are shown (n = 16). Asterisks indicate significant (p ≤ 0.05; *) or highly significant (p ≤ 0.01; **) differences between mean values according to Welch´s t-tests, resp. Games-Howell tests, made separately for each treatment (in case of the differences between non-stressed and stressed plants), resp. for each watering regime (in case of the differences between C, BR1 and BR2 variants).

**Figure 4:**
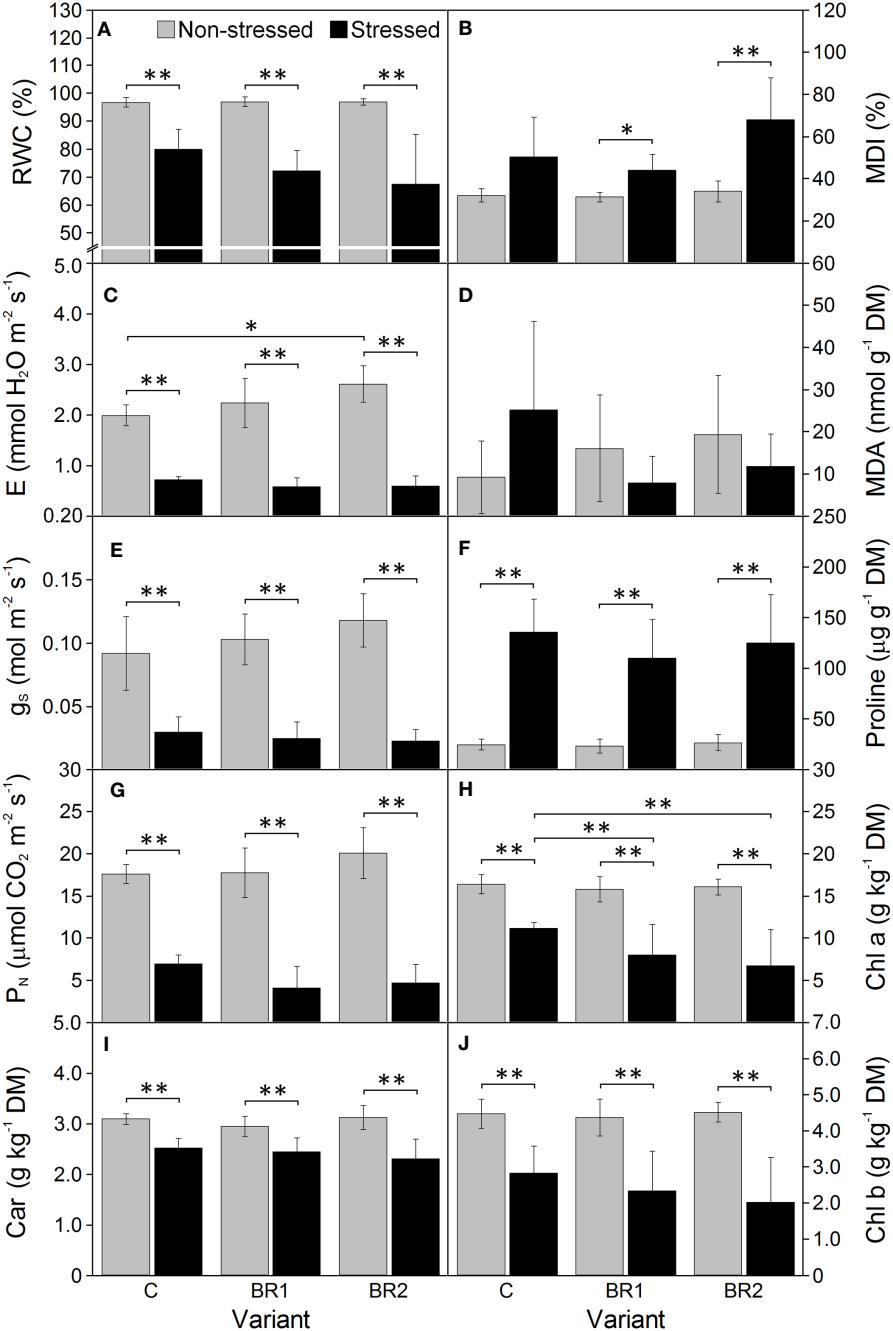
The relative water content **(A)**, the membrane damage index **(B)**, the transpiration rate **(C)**, the malondialdehyde content **(D)**, the stomatal conductance **(E)**, the proline content **(F)**, the net photosynthetic rate **(G)**, the contents of chlorophylls *a* and *b* **(H, J)** and total carotenoids **(I)** measured in maize 3^rd^ leaves at Timepoint 4. Plants were subjected to normal watering (non-stressed plants) or 14 d of withholding water (stressed plants) and treated with 24-*epi*brassinolide (the BR1 and BR2 variants) or with water (the C variant) either before the start of drought period (C, BR1) or during the drought period (BR2). Mean values ± SD are shown (n = 6 for gas exchange parameters, 8-9 for other parameters). For other information see legend to **Figure 3**. DM, dry matter. Asterisks indicate significant (p ≤ 0.05; *) or highly significant (p ≤ 0.01; **) differences between mean values according to Welch´s t-tests, resp.

**Figure 5:**
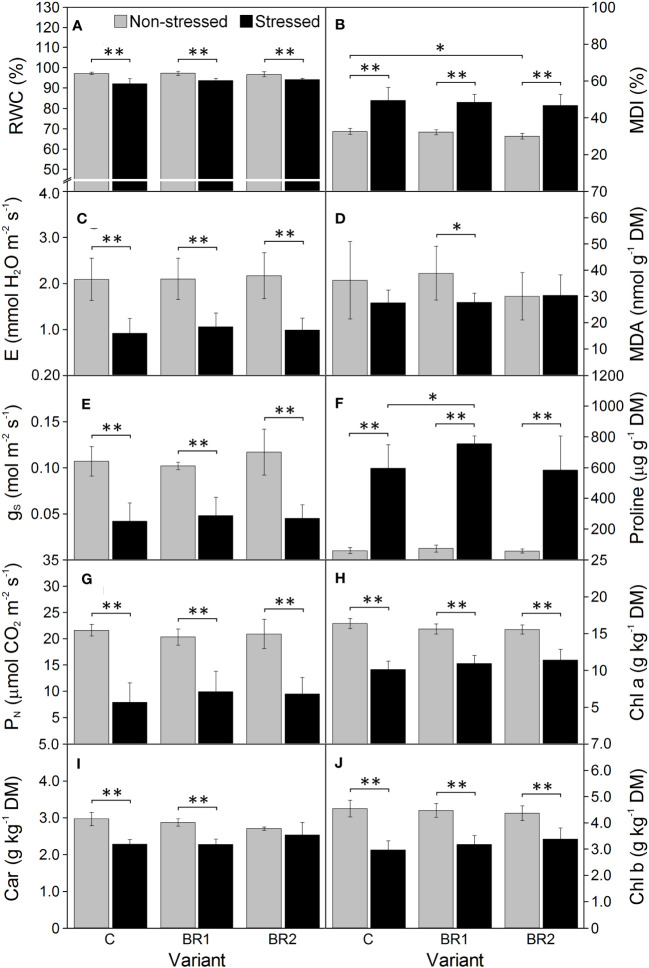
The relative water content **(A)**, the membrane damage index **(B)**, the transpiration rate **(C)**, the malondialdehyde content **(D)**, the stomatal conductance **(E)**, the proline content **(F)**, the net photosynthetic rate **(G)**, the contents of chlorophylls *a* and *b* **(H, J)** and total carotenoids **(I)** measured in maize 4^th^ leaves at Timepoint 4. Plants were subjected to normal watering (non-stressed plants) or 14 d of withholding water (stressed plants) and treated with 24-*epi*brassinolide (the BR1 and BR2 variants) or with water (the C variant) either before the start of drought period (C, BR1) or during the drought period (BR2). Mean values ± SD are shown (n = 6 for gas exchange parameters, 8-9 for other parameters). For other information see legend to **Figure 3**. DM, dry matter. Asterisks indicate significant (p ≤ 0.05; *) or highly significant (p ≤ 0.01; **) differences between mean values according to Welch´s t-tests, resp.

**Figure 6:**
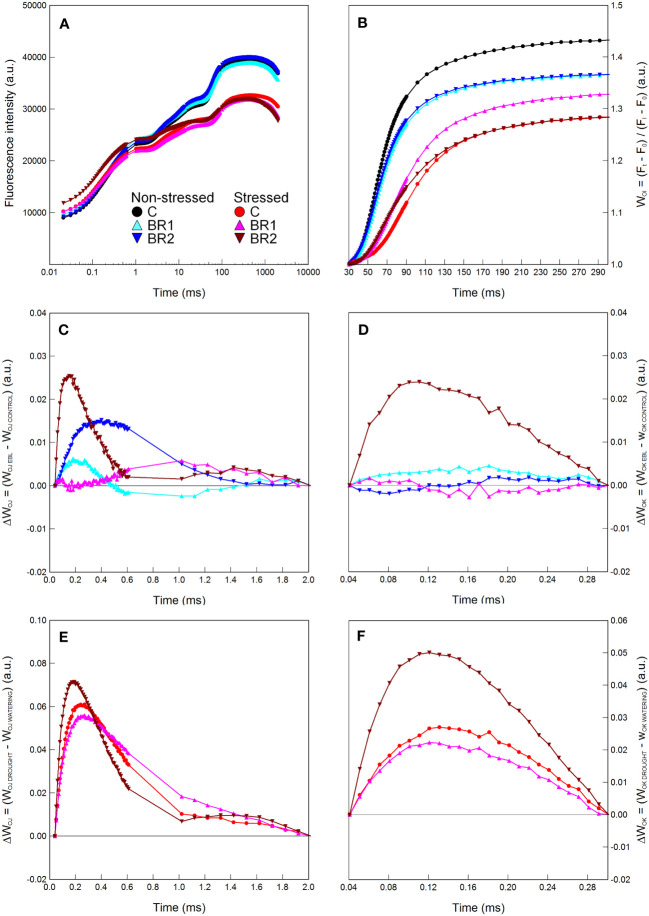
Graphical analysis of the OJIP chlorophyll fluorescence data measured in maize 3^rd^ leaves at Timepoint 4. Plants were either treated with 24-*epi*brassinolide (the BR1 and BR2 variants) or with water (the C variant) either before the start of drought period (C, BR1) or during the drought period (BR2), and subjected to normal watering (non-stressed plants) or 14 d of withholding water (stressed plants). The OJIP curve **(A)**, the relative variable fluorescence W_OI_ **(B)** and the difference kinetics ΔW_OJ_ **(C, E)** and ΔW_OK_ **(D, F)** are shown. ΔW_OJ_ and ΔW_OK_ were calculated either from the comparison of the respective BR1/BR2 and C variants subjected to the same watering regime **(C, D)** or from the comparison of the respective non-stressed and stressed plants subjected to the same type of treatment **(E, F)**. For other information see legend to **Figure 1**.

**Figure 7:**
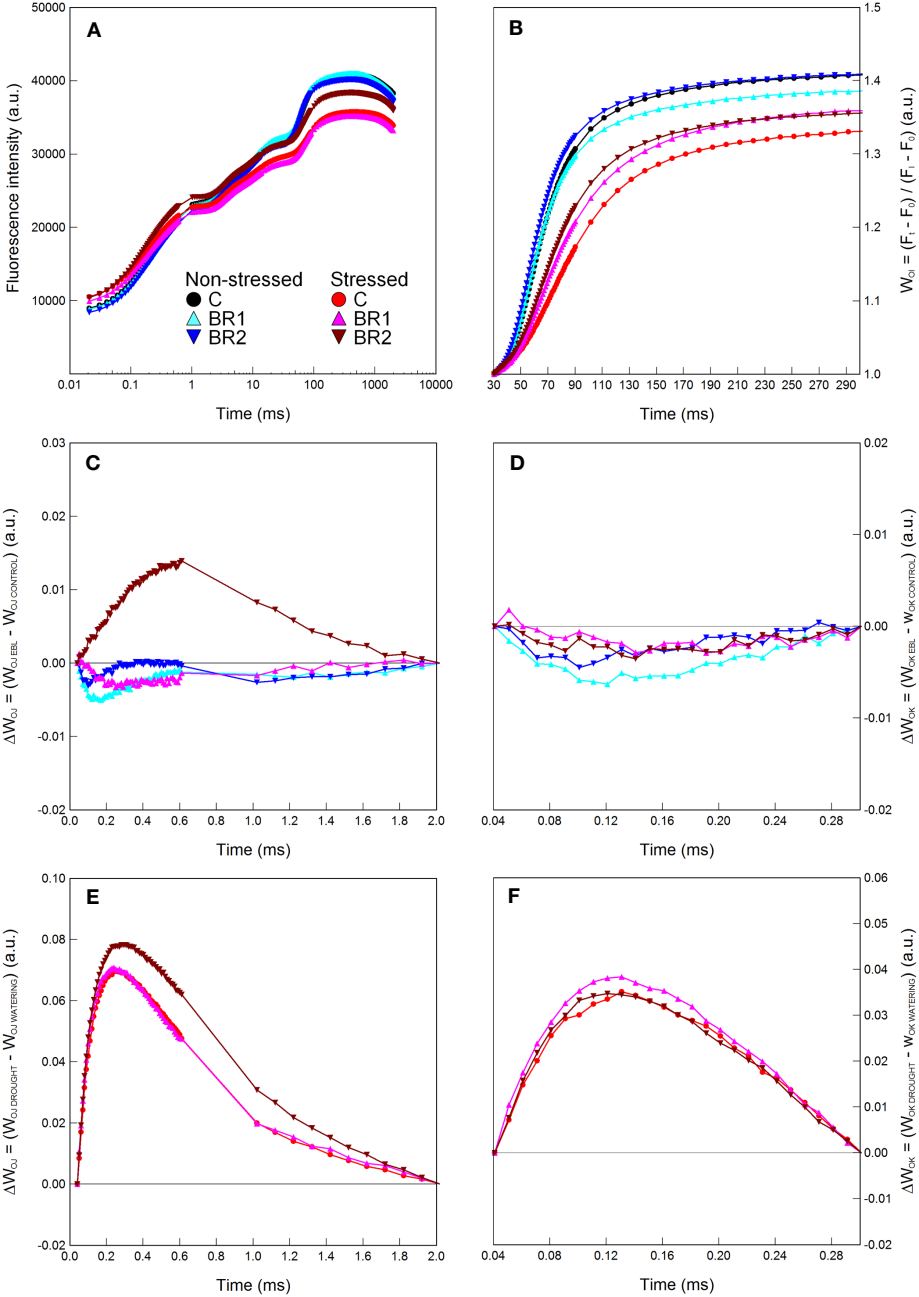
Graphical analysis of the OJIP chlorophyll fluorescence data measured in maize 4^th^ leaves at Timepoint 4. Plants were either treated with 24-*epi*brassinolide (the BR1 and BR2 variants) or with water (the C variant) either before the start of drought period (C, BR1) or during the drought period (BR2), and subjected to normal watering (non-stressed plants) or 14 d of withholding water (stressed plants). The OJIP curve **(A)**, the relative variable fluorescence W_OI_ **(B)** and the difference kinetics ΔW_OJ_ **(C, E)** and ΔW_OK_ **(D, F)** are shown. ΔW_OJ_ and ΔW_OK_ were calculated either from the comparison of the respective BR1/BR2 and C variants subjected to the same watering regime **(C, D)** or from the comparison of the respective non-stressed and stressed plants subjected to the same type of treatment **(E, F)**. For other information see legend to **Figure 1**.

Further drought-induced reduction of the values of all morphological parameters in comparison to the well-watered plants was observed after an additional week of drought simulation with the only exception for the DMR of the C variant (**Figure 3**). The same applied to the RWC and all gas exchange parameters; the reduction of the values of these parameters was more pronounced in the 3^rd^ leaves than in the 4^th^ leaves (**Figures 4**, **5**). The contents of photosynthetic pigments and the efficiency of the PETC also significantly decreased due to drought. In this case, both leaves responded more-or-less similarly, although the most pronounced changes for the 3^rd^ leaves were usually observed in the BR2 variants, whereas for the 4^th^ leaves in the C variants (**Figures 4**–**7**, **Supplementary Table 3**). On the other hand, the exposure of plants to drought caused a significant increase in the dissipation of the excess excitation energy in the PETC (**Supplementary Table 3**) and the MDI values (similar in both leaves, **Figures 4**, **5**). The same applied to the content of proline; the change of this parameter was particularly pronounced in the 4^th^ leaves (**Figures 4**, **5**). We found almost no statistically significant differences in the MDA content between drought-stressed and well-watered plants, probably due to the high biologic variability of the samples (**Figures 4**, **5**).

The BR1 group of well-watered plants showed significantly higher DMR values in comparison to the C group and the number of fully developed leaves was also significantly higher in both *epi*BL-treated groups under these cultivation conditions (**Figure 3**). Regarding the DMS and the plant height, although we could find a slight trend of an *epi*BL-caused increase of the values of these parameters in the well-watered plants, due to the high biologic variability these differences were not statistically significant. Under drought conditions, neither BR1 nor BR2 variants significantly differed from their respective control in either morphological parameter (**Figure 3**).

There were almost no statistically significant differences in the RWC or the gas exchange parameters between our *epi*BL-treated and non-treated plants, although a trend of a slight increase of values of E, g_S_ and P_N_ from the C to the BR2 group could be seen in the 3^rd^ leaves of the well-watered plants (**Figure 4**). Neither there were any significant differences between the *epi*BL-treated and non-treated variants in the contents of photosynthetic pigments. The exception from this was the Chl *a* content in the 3^rd^ leaves of the drought-stressed plants, which was significantly lower in both BR1 and BR2 variants (**Figure 4**). The JIP test parameters describing the efficiency of the PETC also did not show any effect of the *epi*BL treatment either in the well-watered or in the drought-stressed plants (**Supplementary Table 3**). However, the late *epi*BL treatment showed a negative effect on the function of the OEC, as well as on the energetic connectivity among PSII complexes in the 3^rd^ leaves of our drought-stressed plants (**Figure 6**); whereas in the 4^th^ leaves or in the well-watered plants these effects were negligible (**Figure 7**). The size of the pool of end electron acceptors in the PETC was also slightly reduced after both *epi*BL treatments in the 3^rd^ leaves of the well-watered plants, whereas for the drought-stressed plants, the BR1 variant showed a greater size of this pool compared to the C or BR2 variants (**Figure 6**). The differences between individual variants in this respect were less obvious in the 4^th^ leaves (**Figure 7**).

Although the BR1 and BR2 variants exposed to drought showed a reduction of the MDA content in their 3^rd^ leaves in comparison with the respective control, these differences were statistically non-significant due to the high biologic variability of samples (**Figure 4**). No significant differences were found between our *epi*BL-treated and non-treated plants in the MDI (the only exception being the difference between the BR2 and C variants in the 4^th^ leaves of the well-watered plants; **Figure 5**). The proline content was significantly higher in the 4^th^ leaves of the drought-stressed BR1 variant in comparison with the drought-stressed C variant (**Figure 5**).

**Figure 8:**
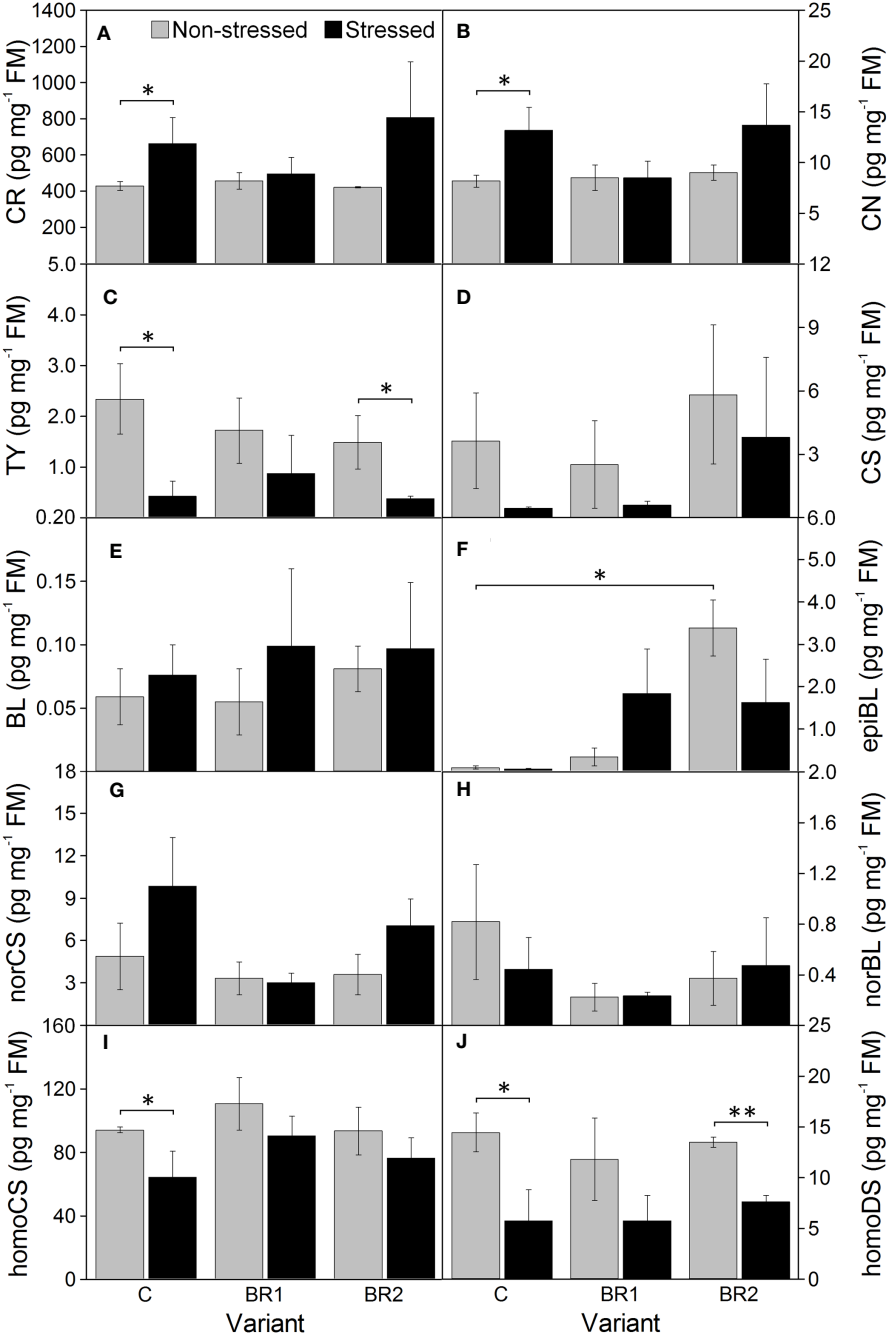
The contents of campesterol **(A)**, campestanol **(B)**, typhasterol **(C)**, castasterone **(D)**, brassinolide **(E)**, 24-*epi*brassinolide **(F)**, 28-*nor*castasterone **(G)**, 28-*nor*brassinolide **(H)**, 28-homocastasterone **(I)** and 28-homodolichosterone **(J)** measured in maize 3^rd^ leaves at Timepoint 4. Plants were subjected to normal watering (non-stressed plants) or 14 d of withholding water (stressed plants) and treated with 24-*epi*brassinolide (the BR1 and BR2 variants) or with water (the C variant) either before the start of drought period (C, BR1) or during the drought period (BR2). Mean values ± SD are shown (n = 4). For other information see legend to **Figure 3**. FM, fresh matter. Asterisks indicate significant (p ≤ 0.05; *) or highly significant (p ≤ 0.01; **) differences between mean values according to Welch´s t-tests, resp.

**Figure 9:**
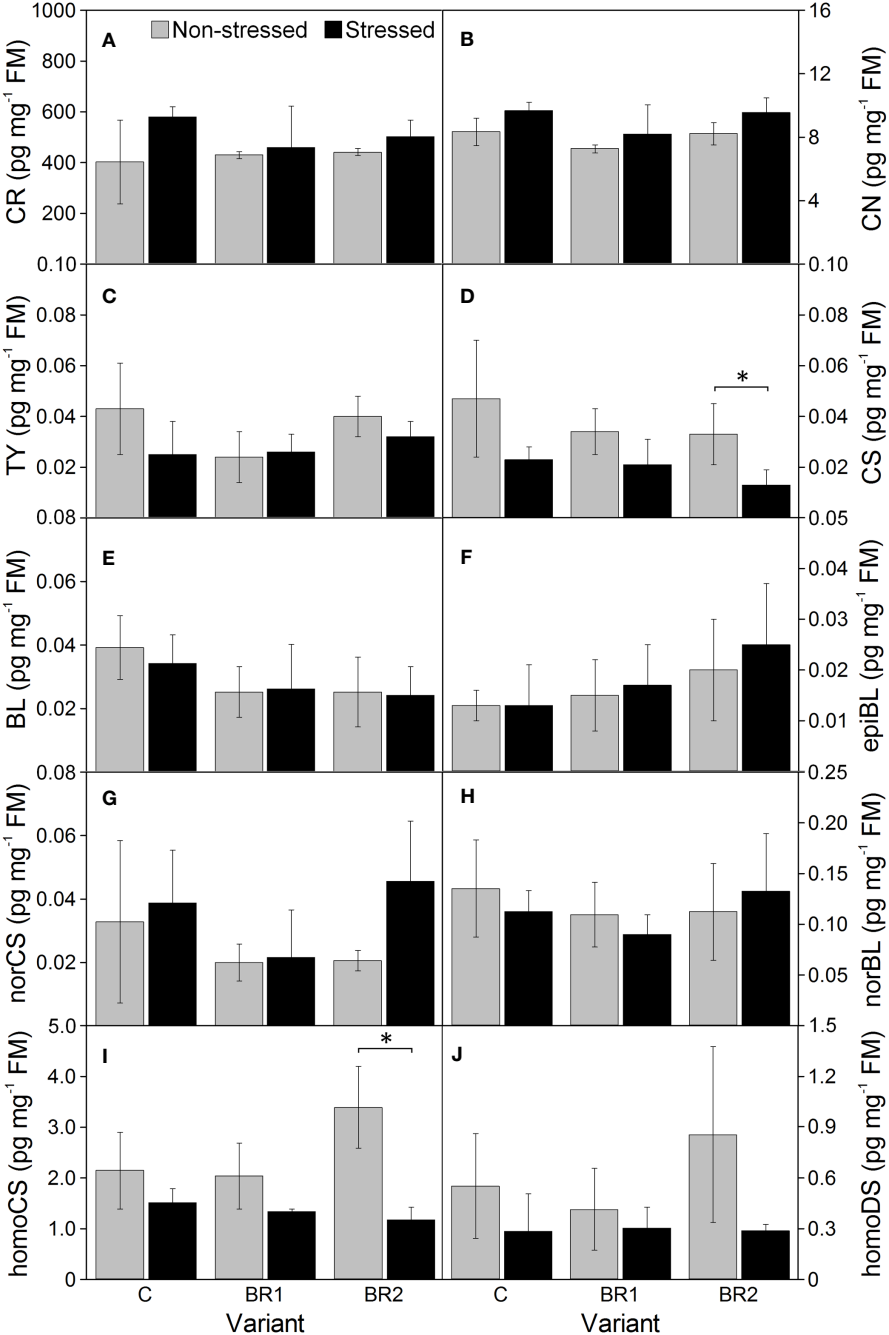
The contents of campesterol **(A)**, campestanol **(B)**, typhasterol **(C)**, castasterone **(D)**, brassinolide **(E)**, 24-*epi*brassinolide **(F)**, 28-*nor*castasterone **(G)**, 28-*nor*brassinolide **(H)**, 28-homocastasterone **(I)** and 28-homodolichosterone **(J)** measured in maize roots at Timepoint 4. Plants were subjected to normal watering (non-stressed plants) or 14 d of withholding water (stressed plants) and treated with 24-*epi*brassinolide (the BR1 and BR2 variants) or with water (the C variant) either before the start of drought period (C, BR1) or during the drought period (BR2). Mean values ± SD are shown (n = 4). For other information see legend to **Figure 3**. FM, fresh matter. Asterisks indicate significant (p ≤ 0.05; *) or highly significant (p ≤ 0.01; **) differences between mean values according to Welch´s t-tests, resp.

**Figure 10:**
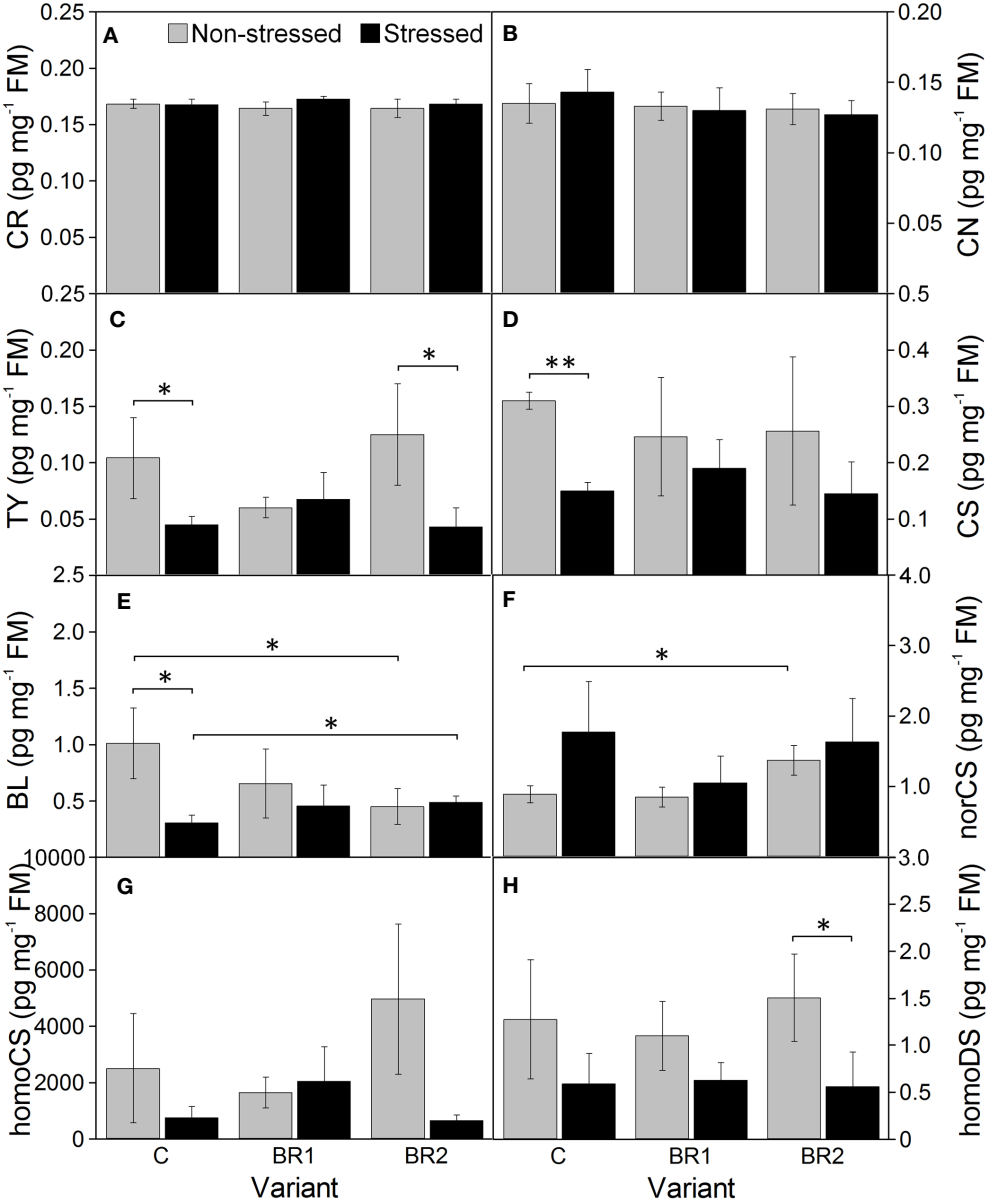
The contents of campesterol **(A)**, campestanol **(B)**, typhasterol **(C)**, castasterone **(D)**, brassinolide **(E)**, 28-*nor*castasterone **(F)**, 28-homocastasterone **(G)**, and 28-homodolichosterone **(H)** measured in maize 4^th^ leaves at Timepoint 4. Plants were subjected to normal watering (non-stressed plants) or 14 d of withholding water (stressed plants) and treated with 24-*epi*brassinolide (the BR1 and BR2 variants) or with water (the C variant) either before the start of drought period (C, BR1) or during the drought period (BR2). Mean values ± SD are shown (n = 4). For other information see legend to **Figure 3**. FM, fresh matter.

Drought caused significant increase of the levels of BR biosynthetic precursors CR and CN in the 3^rd^ leaves of the control plants while this increase was insignificant in root tissue (**Figures 8**, **9**). In the 4^th^ leaves, the levels of these compounds were much lower and no changes due to plant exposure to drought were observed (**Figure 10**). The levels of TY, a direct precursor of bioactive CS, were reduced at the end of the drought simulation period in leaves of the C and BR2 variants (and insignificantly also in roots; **Figures 8**–**10**). The levels of CS also showed a certain reduction, but statistically significant difference was found only for the 4^th^ leaves of the C variant and roots of the BR2 variant, mostly due to the otherwise high variability of samples taken from the well-watered plants (**Figures 8**–**10**). A similar situation was observed for the content of a C_29_-analogue of CS, homoCS, with the exception that a significant difference from the C variant was found in the 3^rd^ leaves (**Figures 8**–**10**). No statistically significant drought-induced changes in the norCS (C_27_-BR) levels were found, although there was a visible trend of the increase of the contents of this compound in the drought-stressed plants of C and BR2 variants compared to the well-watered ones (**Figures 8**–**10**). There were also no statistically significant differences in the BL content between the well-watered and the drought-stressed plants either in the 3^rd^ leaves or in roots (**Figures 8**, **9**). However, drought led to a significant reduction of the content of the most biologically active BR brassinolide in the 4^th^ leaves of plants not treated with *epi*BL, *i.e*., BL (**Figure 10**). Maize was found to be capable for synthesizing 28-homodolichosterone (homoDS) whose levels showed also a reduction as a direct result of drought in both leaves and roots, resp. (**Figures 8**–**10**). The presence of *epi*BL and norBL was detected only in the 3^rd^ leaves and (in much lower amounts) in roots (**Figures 8**, **9**). The levels of these BRs did not significantly differ between well-watered and stressed plants.

The exogenous *epi*BL treatment did not significantly affect the levels of endogenous BRs detected in leaves or roots of our experimental plants (**Figures 8**–**10**). The exception from this were BL levels in the 4^th^ leaves of the BR2 variants, which significantly differed from the C variants regardless of the cultivation conditions (although with opposite trends; **Figure 10**). The 4^th^ leaves of the well-watered BR2 plants had also higher levels of norCS (and, non-significantly, also of homoCS) compared to their controls (**Figure 10**). A statistically significant increase in the *epi*BL levels was observed in the 3^rd^ leaves of the well-watered BR2 variant. There was also an increase in the stressed BR1 and BR2 variants. However, in this case, it was not statistically significant (**Figure 8**).

## 4 Discussion

BRs are generally considered to play many beneficial roles in plant development, physiology and biochemistry, particularly in plants subjected to various sub-optimum conditions. Their exogenous application was shown to improve plant response to both abiotic and biotic stress factors, such as high or low temperature, water deficit or water excess, high or low irradiance, salinity, nutrient deficiency or excess, exposure to heavy metals, herbicides, pesticides, viral or bacterial pathogens, fungi, *etc*. The number of studies dealing with this topic reaches many hundreds and as such, exceeds the possibilities of this paper to be thoroughly discussed. However, although many studies are currently available, various aspects of BR relationship to plant stress response still remain very poorly examined or not explored at all. Our experiments presented in this paper tried to add to our knowledge on some of these poorly understood topics. Thus, in the following paragraphs we will focus only on the five main aspects of our study, as stated in the list of our objectives at the end of the Introduction section. The reader interested in the general topic of BRs in relation to a particular type of plant stress, is referred to recent reviews and book chapters, *e.g.*, (Ahammed et al., 2020; Ahammed et al., 2022), Ramirez and Poppenberger (2020); Basit et al. (2021); Li et al. (2021); Rehman et al. (2022); Sidhu and Bali (2022) and many others.

### 4.1 Effects of exogenous BR application on leaves of different age

One of the aims of our study was to analyze whether the exogenous application of BRs might differently mitigate the negative impact of drought on plants in two different leaves: those that were already fully developed at the beginning of stress and those that were still developing during stress period. Several authors (Mostajeran and Rahimi-Eichi, 2009; Gilgen and Feller, 2014; Budič et al., 2016) found that older leaves are more affected by drought than younger leaves. However, in relation to the BRs role in plant drought response, this aspect has not been previously examined. We exposed 5-weeks old maize plants to gradually induced moderate drought stress, which significantly reduced the values of the majority of the observed parameters associated with plant morphology and physiology. A trend of decreasing values after the exogenous *epi*BL application was found in the older, 3^rd^ leaves, especially for the RWC and parameters characterizing photosynthesis. On the other hand, no such trend was observed for the younger 4^th^ leaves for which we even registered a few signs of BR-induced improvement at the end of our experiments (efficiency of some parts of the PETC and the proline content).

The difference in observed responses of analyzed leaves to BRs application could be connected to BR-induced acceleration of leaf senescence. Several authors (*e.g.*, Sağlam-Çağ, 2007; Bariş and Sağlam-Çağ, 2016; Fedina et al., 2017; Kim et al., 2019) showed that BRs promote leaf senescence (although the exact mechanism is still unknown and might be probably an indirect one; Jan et al., 2019). This is usually accompanied by an increase in membrane damage, reduced Chl contents and decreased photosynthetic efficiency. Thus, the older (3^rd^) leaves of our drought-stressed plants treated with *epi*BL could, particularly at Timepoint 4, already enter the beginning stage of the senescence (reflected in the more pronounced reduction of their Chl content and the PETC efficiency compared to the non-treated stressed plants). Even before the beginning of the drought simulation, the 3^rd^ leaves of plants treated with *epi*BL already showed a worse efficiency of their PETC than the non-treated plants. On the other hand, the 4^th^ leaves were younger so they have not entered the senescence phase even at Timepoint 4 and the BR treatment thus did not affect them negatively. Gomes et al. (2013) also found lower chlorophyll content as a sign of the beginning of senescence in the older drought-stressed papaya leaves after BR treatment but not in the younger ones (this did not apply to the non-stressed plants, similarly to our results). Interestingly, while the later study of these authors made with the same species (Gomes et al., 2018) confirmed that the development of younger leaves does not seem to be particularly accelerated by BRs, leaves that were already mature at the time of BR application showed slightly delayed senescence in BR-treated plants in this case. However, the respective plants were not subjected to any stressor so perhaps it might be the combination of stress and the BR treatment that particularly accelerates leaf senescence.

The expected mitigating effect of BRs on our drought-stressed plants was thus found only in their 4^th^ leaves, but it was very mild and usually statistically non-significant. The reason for this could be that the 4^th^ leaves of our plants did not particularly suffer from drought stress (their RWC values were still above 90% even after 2 weeks of drought simulation). The overwhelming majority of the authors that analyzed the BR/drought relationship by measuring various physiological and biochemical leaf parameters did not state the water status of leaves of their experimental plants at all and those that did usually worked with severely stressed leaves (RWC much below 70% or leaf water potential below -1.5 MPa) (Holá, 2019). It is thus highly probable that there was no opportunity for the *epi*BL treatment to show its potential as a drought-mitigating agens because the 4^th^ leaves of our experimental plants simply did not need it.

The observed change in proline levels in the response to *epi*BL treatment was another interesting phenomenon found only in the 4^th^ leaves of our plants. We found that the *epi*BL application prior to any drought simulation results in significantly reduced proline contents in these leaves, but only in non-stressed plants. Furthemore, this effect did not persist with time: it was observed only during Timepoints 2 and 3, while at Timepoint 4 it faded away and was replaced by a positive effect of *epi*BL pre-treatment on proline levels in the drought-stressed plants. Proline in drought-stressed plants generally functions as an osmoprotectant and its increased accumulation also results in plant protection against oxidative stress (Ghosh et al., 2022). The majority of authors who analyzed the proline content in leaves of drought-stressed plants treated with exogenous BRs (or plants overproducing BRs due to transgenic modification of some BR biosynthetic gene) reported a positive effect of such treatment on this parameter. However, such effects were usually observed only in stressed plants, not in well-watered (control) ones, where BR treatment either did not have any significant effect or even resulted in reduced proline levels (*e.g.*, Zhang et al., 2008; Anjum et al., 2011; Li and Feng, 2011; Talaat et al., 2015; Ahmed et al., 2017; Duan et al., 2017; Khamsuk et al., 2018; Kaya et al., 2019). Although Fu et al. (2019) reported that exogenous *epi*BL pre-treatment significantly elevates the expression of two key proline biosynthetic genes, *P5CS* and *P5CR* (as well as the activities of the respective enzymes and the proline content) in *Elymus nutans* plants, this applied only for plants subjected to cold stress but not for their non-stressed plants. On the other hand, Ábrahám et al. (2003) showed that treatment of non-stressed *Arabidopsis* plants with exogenous *epi*BL inhibits the expression of a *P5CS* gene and thus actually leads to reduced proline levels. It is possible that something similar occurred in our experimental plants. Exogenous BR treatment *per se* certainly does not seem to specifically induce proline biosynthesis (it can even act antagonistically) and it is only in combination with drought (or some other stress factor) that BR application can result in elevated proline levels and thus better osmoprotective and antioxidant parameters of BR-treated plants.

The reason that the effects of *epi*BL application took place only in the younger leaves of our plants is currently unknown. Whether it could be somehow associated with the general differences between younger and more mature (or senescing) leaves in proline levels (which can be different in various plant species, *e.g.*, Cechin et al., 2006; Xue et al., 2008; Sperdouli and Moustakas, 2015), and whether it could be somehow associated with differences in the contents of specific brassinosteroids between these two types of leaves (see below) remains to be seen. The detailed examination of the expression of various genes associated with proline metabolism and/or transport together with a thorough analysis of the activities of the respective proteins in BR-treated, drought-stressed (or non-stressed) plants at different timepoints after the onset of drought and/or BR application, and in leaves in different developmental stage would be certainly worthwhile and while such analysis was outside the scope of our experiments presented in this paper, it is something we would like to focus on in the future.

### 4.2 Effects of exogenous BR application before or during drought

The second aspect we examined in our work was the role of a time when BRs were applied to our experimental plants. We used the *epi*BL application before (BR1 variants) and during (BR2 variants) drought simulation but we found almost no difference in the BR effects on plant physiology and morphology between these two timepoints. The earlier application slightly improved the plant morphology of our non-stressed plants (perhaps because *epi*BL had more time to act on plant growth and development), whereas the later application resulted in slightly higher gas exchange (particularly in their 3^rd^ leaves). However, these differences were mostly negligible and certainly did not apply to the drought-stressed plants. There is very little knowledge on the difference between BR applications before drought and during drought. In two studies available on this topic, the authors used a different type of application and BR concentration for each timepoint, which makes a subsequent comparison inappropriate (Farooq et al., 2009; Hashemi et al., 2015). However, even in these cases, the authors did not find any difference between the effect of BR pre-treatment and post-treatment on their drought-stressed plants. The only marked difference between our drought-stressed BR1 and BR2 variants was that there was a clearly evident more negative effect of *epi*BL applied during the stress period on the performance of the OEC and the excitonic connectivity among individual PSII complexes observed in their 3^rd^ leaves but not in the 4^th^ ones. The primary photosynthetic processes are always more affected by BR treatment in more intensively stressed leaves and the OEC seems to be particularly sensitive to any change in BR homeostasis (Holá, 2022). However, all things considered, it certainly does not seem that the time of exogenous BR application is a particulary important factor that influences the response of drought-stressed plants to these phytohormones.

### 4.3 Effects of exogenous BR application on endogenous C_27_, C_28_-and C_29_- BRs

Our selection of these two separate timepoints for the BR application also meant that the endogenous BR contents were determined in the leaves and roots of our plants at either 17 d (BR1 variants, *i.e.*, plants treated at Timepoint 1) or 7 d (BR2 variants, treated at Timepoint 3) after this application, respectively. Although in most cases the differences in the contents of individual BRs and/or their precursors between BR1/BR2 variants and the non-treated plants were not statistically significant, there was a clearly pronounced trend of a higher increase of the amounts of C_29_-BRs (homoCS, homoDS) in the 4^th^ leaves and roots of the non-stressed BR2 variant. For norCS, which is a representative of C_27_-BRs, this increase in the 4^th^ leaves was even statistically significant, whereas the content of BL (a representative of C_28_-BRs) significantly increased under stress conditions (particularly after the later BR application) but showed an opposite trend in our well-watered plants. These observed trends suggest that the changes in the levels of endogenous BRs caused by the exogenous BR application probably diminish with time (*e.g.* due to metabolization – see Schneider et al., 1994; Kolbe et al., 1995) and that the shorter period between the BR treatment and the analysis of endogenous BR contents is better for them to be detectable. This is supported also by our observation that the contents of *epi*BL in the 3^rd^ leaves of our plants significantly increased in the order of C < BR1 < BR2, following the decreasing length of the period between *epi*BL spraying and BR content determination. We think that the detected *epi*BL levels in our BR1 and BR2 variants could be residues after the respective *epi*BL treatments, which persisted in a tissue of interest to the last sampling point. A similar situation was described for another monocot (wheat) plant by Janeczko and Swaczynová (2010). The absence of *epi*BL in the 4^th^ leaves could be explained either by the fact that these leaves were yet rather small during the spraying and thus offered less area for BR entry (the BR1 variant), or by the possibility that these leaves could use a completely different mechanism how to deal with exogenously applied BRs. For instance, Nishikawa et al. (1995) showed that while hypocotyls and roots of cucumber are not much able to metabolize *epi*BL, petioles and leaves do have this ability. A similar situation was observed by the same research team in intact wheat seedlings. Thus, there might be some differences in the metabolization of particular exogenous substance in various plant parts/organs in this respect.

The effect of the exogenous BR application on the endogenous BR contents depends not only on *when* but also on *how* BRs are applied, as well as on plant species and BR concentration. We utilized foliage spraying as it is the most frequently employed method in BR/drought studies. However, the majority of information available on the possible changes in endogenous BR contents caused by the exogenous BR application comes from studies where BRs were either added directly into the growth medium and plants received them through roots, or by soaking of seeds in BR solutions (**Supplementary File 2**). Only Janeczko and Swaczynová (2010) sprayed wheat plants in the V2 stage with *epi*BL solutions and they did not observe any changes in the CS or BL contents in the 3^rd^ leaves of their plants 11 d after spraying. Janeczko et al. (2011) also used direct injection of *epi*BL into the 1^st^ and 2^nd^ leaves of barley. Using this approach, they did not found any significant effect on endogenous CS or BL contents in the 7^th^ leaf after 20 d. Notably, both CS and BL belong to the C_28_-BR group and none of these authors analyzed C_27_- or C_29_-BRs in their plants at that time. In later studies, some authors that applied BRs directly into the growth medium or by seed soaking also observed an increase in the levels of C_27_- or C_29_-BRs but not C_28_-BRs (Filek et al., 2019; Tarkowská et al., 2020) while others found that such type of application can elevate the contents of C_28_-BRs as well (depending on the concentration and/or plant species and analyzed plant organ) (Bajguz et al., 2019; Chmur and Bajguz, 2021). It seems that while the application of BRs into growth medium, particularly in hydroponic cultures, can evidently affect their contents in leaves/shoots (either by enabling their direct transport through a plant as suggested by Janeczko and Swaczynová, 2010, or, more probably, by an indirect effect on the regulation of BR biosynthesis), the direct treatment of leaves/shoots (as in our case) seems to have a more negligible effect on the endogenous BR levels. This is probably due to the fact that BRs applied in this manner are not transported over long distances (Symons and Reid, 2004; Symons et al., 2008) and their effect is only temporary and diminishes with time.

### 4.4 Effects of analyzed organ and leaf age on endogenous C_27_, C_28_-and C_29_-BRs

The biosynthetic precursors of C_29_-BRs (as well as the respective end products in monocots) are frequently found to be present in much higher amounts compared to C_28_-BRs in various plant species that have been examined to this date (**Supplementary File 2**). This is in good agreement with our findings: both the 3^rd^ and 4^th^ leaves and roots of our plants contained more homoCS and homoDL than BRs belonging to the C_28_ group. When the levels of BRs and/or their precursors in leaves and roots of our experimental plants were compared, we also found a good agreement between our data and the majority of data published previously by authors who simultaneously analyzed endogenous BR levels in shoots/leaves and roots of various plant species (**Supplementary File 2**). The contents of CS and BL are consistently reported to be higher in the aboveground vegetative parts of plants than in roots and our results supports this observation as well. The contents of TY were found by some authors to be also higher in leaves than in roots (Jiang et al., 2012, in rice, Pavlović et al., 2019, in Chinese cabbage, Lu et al., 2021 in sunflower), which also corresponds to our observations and to the results of earlier reports describing the lowest levels of BRs in root tissue (Bancos et al., 2002; Shimada et al., 2003; Symons and Reid, 2004). In contrast, there are some other authors who reported either similar levels of TY in shoots and roots or even TY elevation in roots (**Supplementary File 2**). Altogether it seems that the differences in the TY content between shoots/leaves and roots might depend on plant species or some other experimental aspects. A similar discrepancy of data can be observed also for CR and CN: while we found comparable amounts of these C_28_-BR precursors in the 3^rd^ leaves and roots of our maize plants (much lower amounts were detected in the 4^th^ leaves) and Nakamura et al. (2006) reported higher levels of CR and CN in rice shoots than in roots, the opposite situation was found by Shimada et al. (2003) and Kim et al. (2006) for *Arabidopsis*. Regarding C_27_- and C_29_-BRs, our data were again in good agreement with the findings of Yokota et al. (2001) and Luo et al. (2018), who reported lower levels of the end products of the respective biosynthetic pathways (or even the complete absence of these BRs) in roots of tomato and rice as compared to shoots.

The situation with the BR contents in the 3^rd^ and the 4^th^ leaf is more interesting than the simple comparison of BR quantity in leaves and roots. Actually, we think that this is probably our most interesting finding presented in this study. The aspect of the comparison of a profile of BRs belonging to various structural groups in younger and more mature leaves has not been much dealt with. When searching the available literature, we found only two studies discussing the content and portfolio of C_27_- to C_29_-BRs in the leaves of different ages. Zhang et al. (2022a) reported a slightly higher content of CS (a C_28_-BR) for older leaves compared with younger leaves of their tea (*Camellia sinensis)* plants. This is in agreement with our own findings, although in our case, the difference between leaves was of a much higher order of magnitude. On the other hand, the amount of norCS (a C_27_-BR, which is synthesized mostly from 6-oxocholestanol with cholesterol and cholestanol as earlier precursors (Bajguz et al., 2020) was lower in the 4^th^ (younger) leaves of our plants, whereas Zhang et al. (2022a) did not report any differences. The second study published on this topic is that of Parada et al. (2022) using a grapevine (*Vitis vinifera*) as a model plant. In their case, CS and homoDS (C_28_- and C_29_-BRs, resp.) were detected in neither young nor mature leaves, *epi*CS was present in the young leaves only and the level of norTE and homoCS (C_27_- and C_29_-BRs, resp.) was higher in the mature leaves compared to younger ones. This is inconsistent with our results where the levels of homoCS are about an order of magnitude higher in our younger leaves (4^th^) than in the older leaves (3^rd^).

Our above-mentioned data lead strongly to the suggestion that the younger, 4^th^ leaves of maize probably for some reason divert their BR biosynthesis from C_28_-BRs (TY, CS, BL, *epi*BL) to C_29_-BRs like homoCS. The biosynthesis of C_27_-, C_28_- and C_29_-BRs is interconnected at various levels. CS can be synthesized from many C_27_- to C_29_-BRs precursors including norCS, homoCS and homoDS (Bajguz et al., 2020). However, we think that the divergence of BR biosynthesis in our younger leaves probably occurs at the very early biosynthetic steps, prior to the beginning of the late C-22 oxidation pathway, because the 4^th^ leaves appeared to be a very poor source of CR and CN. Thus, the BR biosynthesis in the younger maize leaves (at least in this inbred line) is probably strongly redirected from the CR (and/or cholesterol) branches to the parallel sitosterol branch of the BR biosynthetic pathway (Bajguz et al., 2020; Wei and Li, 2020). This might explain the highly elevated level of homoCS then. With respect to the levels of the homoDS, one can only speculate that *within* the sitosterol branch the biosynthesis is not further diverted *via* 22*S*-hydroxyisofucosterol, which is proposed to be a precursor of homoDS (Bajguz et al., 2020).

### 4.5 Effects of drought on endogenous C_27_, C_28_-and C_29_-BRs

The final part of our experiment to be discussed is the effect of drought *per se* on the levels of individual BRs and their precursors. We found that drought slightly enhanced amounts of CR and CN and reduced amounts of TY, homoCS and homoDS in the leaves (and to a less extent in roots) of our experimental plants. The reduction of homoCS levels was particularly pronounced in the 4^th^ leaves. Drought also led to reduced levels of CS in the 3^rd^ leaves (in the 4^th^ leaves there were mostly no changes or even an increase), while for BL the situation was usually the opposite. Regarding C_27_-BRs, exposure of plants to drought was associated with generally higher amounts of norCS but mostly no changes of the norBL content. The observed changes in the levels of individual BRs are generally consistent with the results of our previous study made with the same inbred line of maize (Tůmová et al., 2018). Similarly to that study, we also did not detect the presence of either DS, DL, homoBL, homoDL or norTE. The unchanged levels of CS in our 4^th^ leaves are also in agreement with previous studies on drought-stressed young pea leaves (Jager et al., 2008), tobacco leaves (Duan et al., 2017) or rice shoots (Ding et al., 2014). Additionally, in the last study, BL was detected in well-watered plants but not in the drought-stressed ones.

As stated above, CR and CN are precursors in the late C-22 oxidation pathway leading to the biosynthesis of the C_28_-BRs (Bajguz et al., 2020). Their slightly increased accumulation in our drought-stressed plants together with the reduced levels of the products of the subsequent early C-6 oxidation pathway (TY, CS) could indicate at least partial inhibition of this BR biosynthetic pathway after plant exposure to drought. However, CS can be also synthesized from homoCS and homoDS (Bajguz et al., 2020) and the reduced levels of these two BRs in our plants suggest the association of drought exposure not only with the inhibition of the C_28_-BRs biosynthetic pathway but also with the reduced biosynthesis of C_29_-BRs. Alternatively, CS can be also synthesized from norCS (Bajguz et al., 2020), and here the observed reduction of CS amounts in our 3^rd^ leaves together with the accumulation of norCS could perhaps mean that the C_27_-biosynthesis *per se* is not inhibited by drought in maize but that the conversion of norCS to CS is. Thus, we suggest that drought inhibits C_28_-BRs and C_29_-BRs biosynthetic pathways whereas C_27_-BRs biosynthesis is not particularly affected. Further, the inhibition of the biosynthesis of C_28_-BRs applies particularly for the older leaves whereas the inhibition of C_29_-BR biosynthesis is more pronounced in the younger ones (which can be associated with the above-mentioned preferences for one or the other biosynthetic pathway in these two types of leaves).

It is worth emphasizing that this endogenous BR response to drought in maize might not be a universal response and does not have to apply to other plant species (or even cultivars of the same species). Even closely related species can show very different responses of their endogenous BR contents to drought as demonstrated by Pavlović et al. (2018) who analyzed three species of *Brassica* under control and drought conditions. They found a reduction of TY amounts, an elevation of CS amounts, and an even more significant elevation of BL amounts in their stressed Chinese cabbage plants whereas there were no changes in the other two species except a mild reduction of BL levels in kale and a mild increase of TY levels in white cabbage (Pavlović et al., 2018). Unfortunately, scientists who studied endogenous BR contents in plants under drought conditions usually analyzed only several (or only one) C_28_-BRs and no C_27_- or C_29_-BRs (**Supplementary File 2**). Only Gruszka et al. (2016) found increased CS levels and mostly unchanged homoBL levels in barley exposed to the insufficient water supply as compared to the well-watered plants. On the other hand, Malaga et al. (2020) reported mostly no changes for CS but a significant increase of homoCS amounts in leaves of some drought-sensitive barley cultivars but not in cultivars with moderate or high drought tolerance. Our previous study with maize showed intraspecific differences in drought-induced changes of endogenous BR levels as well: the drought-tolerant inbred line had been analyzed together with the drought-sensitive one and differed in the contents of some individual BRs (particularly TY, norCS, norBL) both under normal and drought conditions (Tůmová et al., 2018). Thus, to better understand the relationship between drought and biosynthesis/metabolism of various BRs, more detailed analyses of more plant species, more cultivars of the same species, made under mild, moderate, or severe drought conditions, and including plants/leaves of different developmental stages are clearly very much needed.

## 5 Conclusions

The results of our experiments presented in this paper offered several interesting answers to the five main objectives of our study. Firstly, when determining the response of these two types of leaves to the combination of drought exposure and the application of exogenous *epi*BL, we found that the response of leaves of different ages differs: the older leaves can show accelerated senescence under such conditions reflected in their reduced chlorophyll content and diminished efficiency of the primary photosynthetic processes, whereas the younger leaves are characterized by interesting changes of proline levels in response to *epi*BL treatment (this deserves a further, more detailed exploration). Secondly, the exogenous application of *epi*BL before or during drought did not result in a different response of plants to this stress factor (which disproved our original hypothesis on this topic). Thirdly, we showed that the contents of C_27_- and C_29_-BRs in plants treated with exogenous *epi*BL depended on the length of time between this treatment and the BR analysis (which confirmed our original hypothesis) Finally, we demonstrated marked differences in the contents of individual BRs between younger and older maize leaves, which is, in our opinion, the most interesting result of our study. We propose that the younger maize leaves divert their BR biosynthesis from C_28_-BRs to C_29_-BRs, probably at the very early biosynthetic steps (prior to the beginning of the late C-22 oxidation pathway). Drought also apparently negatively affected biosynthetic pathways of C_28_-BRs (particularly in the older leaves) and C_29_-BRs (particularly in the younger leaves) but not C_27_-BRs in our maize plants.

## Data availability statement

The original contributions presented in the study are included in the article/**Supplementary Material**. Further inquiries can be directed to the corresponding author.

## Author contributions

HM and DH conceived the project, DT performed endogenous brassinosteroids measurements, HM and PČ carried out other biochemical analyses, MK and OR performed *in situ* measurements and samplings. HM, DH and DT drafted the manuscript and all authors reviewed and approved the manuscript. All authors contributed to the article and approved the submitted version.

## Supplementary material

Supplementary File 1 The original data on the brassinosteroid contents, plant morphology and various physiological and biochemical characteristics measured at four different timepoints in the 3^rd^ and 4^th^ leaves (or roots) of maize subjected to normal watering or withholding water and treated with 24-*epi*brassinolide (the BR1 and BR2 variants) or with water (the C variant) either before the start of drought period (C, BR1) or during the drought period (BR2).

Supplementary File 2 Heat maps showing the approximate contents of various brassinosteroids (BRs) and their precursors in the non-reproductive parts/organs of various plant species as reported from previously published studies. Only studies dealing with *i)* the effect of exogenous BR application on their endogenous contents; *ii)* the analysis of at least some BRs from both C_28_-, C_29_- and/or C_27_-groups at the same time; *iii)* the analysis of BR contents in both shoots/leaves and roots at the same time, or *iv)* the analysis of BR contents in plants subjected to drought are included here.
