# Supplementary material for: Contents of endogenous brassinosteroids and the response to drought and/or exogenously applied 24-epibrassinolide in two different maize leaves
Source: Front Plant Sci. 2023 Jun 2;14:1139162. doi: 10.3389/fpls.2023.1139162 (PMC10272441; doi:10.3389/fpls.2023.1139162)

Supplementary Material

**Contents of endogenous brassinosteroids and the response to drought and/or exogenously applied 24-*epi*brassinolide in two different maize leaves**

**Hana Marková, Danuše Tarkowská, Petr Čečetka, Marie Kočová, Olga Rothová, Dana Holá***

*** Correspondence:** Dana Holá: danahola@natur.cuni.cz

**Supplementary Table 1.** Selected photosynthetic parameters of the JIP test derived from the measurements of the polyphasic rise of chlorophyll *a* fluorescence transient. F_0_ - the initial fluorescence intensity (at 40 µs), F_K_ - the fluorescence intensity at the K-step (at 300 µs), F_J_ - the fluorescence intensity at the J-step (at 2 ms), F_I_ - the fluorescence intensity at the I-step (at 30 ms), F_M_ ≈ F_P_ - the maximum fluorescence intensity, PSI - photosystem I, PSII - photosystem II, RC - reaction center.

| Parameter | Definition, biologic meaning | Formula |
| --- | --- | --- |
| V_J_ | Relative variable fluorescence at the J-step | (F_J_-F_0_)/(F_M_-F_0_) |
| V_I_ | Relative variable fluorescence at the I-step | (F_I_-F_0_)/(F_M_-F_0_) |
| M_0_ | Approximated initial slope of the fluorescence transient | 4(F_K_-F_0_)/(F_M_-F_0_) |
| φ_P0_ | Maximum quantum yield of primary PSII photochemistry | (F_M_-F_0_)/F_M_ |
| φ_E0_ | Quantum yield of electron transport flux from Q_A_ to Q_B_ | [1-(F_0_/F_M_)](1-V_J_) |
| φ_RE01_ | Quantum yield of electron transport flux until the PSI electron acceptors | 1-(F_I_/F_M_) |
| φ_D0_ | Quantum yield of energy dissipation | F_0_/F_M_ |
| ψ_E0_ | Efficiency/probability with which a PSII trapped electron is transferred from Q_A_ to Q_B_ | 1-V_J_ |
| ψ_RE01_ | Efficiency/probability with which a PSII trapped electron is transferred until PSI acceptors | 1-V_I_ |
| δ_RE01_ | Efficiency/probability with which an electron from Q_B_ is transferred until PSI acceptors | (1-V_I_)/(1-V_J_) |
| γRC2 | Probability that a PSII chlorophyll functions as RC | 1/(ABS/RC+1) |
| ABS/RC | Average absorbed photon flux per PSII RC (apparent antenna size of an active PSII) | (M_0_/V_J_)(1/φ_P0_) |
| TP_0_/RC | Maximum trapped exciton flux per PSII | M_0_/V_J_ |
| ET_0_/RC | Electron transport flux from Q_A_ to Q_B_ per PSII | (M_0_/V_J_)ψ_E0_ |
| RE_01_/RC | Electron transport flux until PSI acceptors per PSII | (M_0_/V_J_)ψ_RE01_ |
| DI_0_/RC | Dissipated energy flux per PSII | (ABS/RC)-(TP_0_/RC) |
| PI_ABS_ | Performance index for energy conservation from photons absorbed by PSII antenna, to the reduction of Q_B_ | [1/(ABS/RC)][φ_P0_/(1-φ_P0_)][ψ_E0_/(1-ψ_E0_)] |
| PI_TOTAL_ | Performance index for energy conservation from photons absorbed by PSII antenna, until the reduction of PSI acceptors | PI_ABS_[δ_RE01_/(1-δ_RE01_)] |

**Supplementary Table 2.** Selected morphological, physiological and biochemical parameters measured at Timepoint 2 in maize leaves (for the biologic meanings of the JIP test parameters see Supplementary Table 1). Plants were subjected to normal watering and treated with 24-*epi*brassinolide (the BR1 variant) or with water (the C variant). Statistically significant differences (p ≤ 0.05) between the respective C and BR1 variants according to Welch´s t-tests are shown in bold.

|  | 3^rd^ leaf *(or the whole plant *)* | | 4^th^ leaf | |
| --- | --- | --- | --- | --- |
|  | C | BR1 | C | BR1 |
| Plant height (mm) * | 130.81±20.82 | 134.56±21.40 |  |  |
| Number of leaves * | 3.38±0.50 | 3.25±0.45 |  |  |
| DMS (g) * | 0.84±0.37 | 0.96±0.34 |  |  |
| DMR (g) * | 0.35±0.13 | 0.40±0.11 |  |  |
| RWC (%) | 96.68±1.08 | 96.72±1.79 | 96.15±3.18 | 98.12±1.05 |
| E (mmol H_2_O m^-2^ s^-1^) | 2.67±0.60 | 2.61±0.22 | 1.89±0.43 | 2.01±0.31 |
| g_S_ (mol m^-2^ s^-1^) | 0.11±0.01 | 0.11±0.02 | 0.11±0.02 | 0.12±0.02 |
| P_N_ (μmol CO_2_ m^-2^ s^-1^) | 23.79±2.44 | 22.69±3.86 | 22.59±2.70 | 23.60±2.33 |
| Chl *a* content (g kg^-1^) | 17.06±0.47 | 17.83±1.45 | 14.70±0.35 | 14.86±0.82 |
| Chl *b* content (g kg^-1^) | 4.42±0.51 | 4.55±0.51 | 3.98±0.34 | 4.05±0.20 |
| Car content (g kg^-1^) | 3.29±0.24 | 3.35±0.31 | 2.60±0.08 | 2.73±0.16 |
| MDI (%) | 33.19±3.19 | 33.44±4.28 | 34.34±2.75 | 32.08±2.64 |
| MDA content (nmol g^-1^) | 20.29±4.12 | 16.07±17.10 | 42.79±6.38 | 32.42±12.80 |
| Proline content (mg g^-1^) | 29.38±4.96 | 31.08±6.50 | **67.43±26.47** | **36.12±12.92** |
| φ_P0_ | 0.79±0.02 | 0.79±0.01 | 0.78±0.03 | 0.78±0.02 |
| φ_E0_ | 1.40±0.03 | 1.41±0.02 | 1.34±0.02 | 1.31±0.04 |
| φ_RE01_ | 0.25±0.02 | 0.24±0.02 | 0.28±0.02 | 0.29±0.02 |
| φ_D0_ | 0.21±0.01 | 0.21±0.01 | 0.22±0.03 | 0.22±0.02 |
| ψ_E0_ | 0.56±0.01 | 0.56±0.02 | 0.58±0.01 | 0.59±0.01 |
| ψ_RE01_ | 0.32±0.02 | 0.30±0.02 | 0.36±0.03 | 0.37±0.04 |
| δ_RE01_ | 0.57±0.03 | 0.54±0.03 | 0.62±0.06 | 0.63±0.06 |
| γRC2 | 0.58±0.02 | 0.57±0.01 | 0.57±0.02 | 0.57±0.02 |
| ABS/RC | 0.72±0.05 | 0.74±0.03 | 0.75±0.05 | 0.75±0.05 |
| TP_0_/RC | 0.57±0.03 | 0.60±0.03 | 0.59±0.02 | 0.58±0.02 |
| ET_0_/RC | 0.32±0.02 | 0.33±0.01 | 0.34±0.01 | 0.34±0.01 |
| RE_01_/RC | 1.76±0.19 | 1.97±0.21 | 1.65±0.18 | 1.59±0.16 |
| DI_0_/RC | 0.15±0.02 | 0.16±0.01 | 0.17±0.03 | 0.17±0.03 |
| PI_ABS_ | 7.02±0.56 | 6.24±1.24 | 6.76±1.34 | 6.78±1.45 |
| PI_TOTAL_ | 9.16±1.96 | 7.55±2.04 | 10.90±2.30 | 11.61±3.11 |

**Supplementary Table 3.** Selected JIP test parameters measured at Timepoint 4 in maize leaves (for their biologic meanings see Supplementary Table 1). Plants were subjected to normal watering (well-watered plants) or 14 d of withholding water (drought-stressed plants) and treated with 24-*epi*brassinolide (the BR1 and BR2 variants) or with water (the C variant) either before the start of drought period (C, BR1) or during the drought period (BR2). Statistically significant differences (p ≤ 0.05) according to Welch´s t-tests between the respective well-watered and drought-stressed variants treated with the same solution are shown in italics. No statistically significant differences between the respective C and BR1 or BR2 variants subjected to the same cultivation conditions were found.

|  | 3^rd^ leaf | | | | | | 4^th^ leaf | | | | | |
| --- | --- | --- | --- | --- | --- | --- | --- | --- | --- | --- | --- | --- |
|  | Well-watered plants | | | Drought-stressed plants | | | Well-watered plants | | | Drought-stressed plants | | |
|  | C | BR1 | BR2 | C | BR1 | BR2 | C | BR1 | BR2 | C | BR1 | BR2 |
| φ_P0_ | *0.75±0.03* | *0.74±0.04* | *0.75±0.03* | *0.65±0.05* | *0.67±0.05* | *0.59±0.10* | *0.76±0.06* | 0.77±0.06 | *0.77±0.02* | *0.67±0.07* | 0.67±0.11 | *0.70±0.04* |
| φ_E0_ | 1.38±0.07 | 1.40±0.12 | 1.39±0.05 | 1.35±0.08 | 1.42±0.07 | 1.66±0.37 | *1.38±0.02* | 1.38±0.06 | *1.39±0.02* | *1.31±0.09* | 1.33±0.12 | *1.35±0.05* |
| φ_RE01_ | *0.23±0.04* | 0.19±0.05 | *0.20±0.03* | *0.15±0.03* | 0.16±0.04 | *0.13±0.05* | *0.22±0.04* | 0.21±0.04 | *0.22±0.02* | *0.17±0.04* | 0.18±0.03 | *0.19±0.02* |
| φ_D0_ | *0.25±0.03* | *0.26±0.04* | *0.25±0.03* | *0.35±0.05* | *0.33±0.05* | *0.41±0.10* | *0.24±0.06* | 0.23±0.06 | *0.23±0.02* | *0.33±0.07* | 0.33±0.11 | *0.30±0.04* |
| ψ_E0_ | *0.54±0.02* | 0.53±0.05 | *0.54±0.03* | *0.47±0.02* | 0.45±0.11 | *0.38±0.14* | 0.55±0.03 | 0.55±0.02 | *0.55±0.02* | 0.52±0.04 | 0.50±0.06 | *0.52±0.02* |
| ψ_RE01_ | *0.30±0.06* | 0.27±0.06 | 0.27±0.04 | *0.22±0.04* | 0.26±0.05 | 0.22±0.05 | 0.29±0.03 | 0.28±0.04 | 0.29±0.03 | 0.25±0.04 | 0.27±0.02 | 0.26±0.03 |
| δ_RE01_ | 0.56±0.09 | 0.52±0.08 | 0.50±0.07 | 0.49±0.09 | 0.50±0.10 | 0.62±0.14 | 0.53±0.06 | 0.50±0.05 | 0.53±0.04 | 0.49±0.06 | 0.55±0.11 | 0.51±0.05 |
| γRC2 | *0.54±0.02* | *0.53±0.04* | *0.54±0.02* | *0.49±0.01* | *0.49±0.03* | *0.45±0.05* | *0.56±0.03* | *0.57±0.03* | *0.57±0.02* | *0.50±0.04* | *0.50±0.06* | *0.51±0.02* |
| ABS/RC | *0.85±0.07* | 0.89±0.14 | *0.87±0.08* | *1.06±0.09* | 1.04±0.13 | *1.24±0.27* | *0.78±0.11* | 0.77±0.12 | *0.77±0.06* | *0.99±0.16* | 1.03±0.30 | *0.96±0.10* |
| TP_0_/RC | *0.63±0.03* | *0.63±0.04* | *0.64±0.04* | *0.69±0.02* | *0.68±0.03* | *0.69±0.03* | *0.59±0.04* | *0.59±0.04* | *0.59±0.03* | *0.66±0.04* | *0.66±0.04* | *0.67±0.03* |
| ET_0_/RC | 0.34±0.01 | 0.33±0.03 | 0.35±0.01 | 0.34±0.02 | 0.34±0.03 | 0.26±0.09 | 0.32±0.02 | 0.32±0.01 | *0.33±0.01* | 0.34±0.02 | 0.33±0.03 | *0.35±0.02* |
| RE_01_/RC | *2.15±0.45* | 2.51±0.72 | *2.31±0.30* | *3.15±0.56* | 2.82±0.20 | *3.37±0.89* | *2.07±0.37* | 2.14±0.39 | *2.04±0.26* | *2.65±0.48* | 2.43±0.16 | *2.55±0.33* |
| DI_0_/RC | *0.22±0.04* | *0.22±0.06* | *0.22±0.04* | *0.37±0.08* | *0.35±0.10* | *0.53±0.23* | *0.19±0.08* | 0.19±0.08 | *0.18±0.03* | *0.33±0.13* | 0.37±0.27 | *0.29±0.07* |
| PI_ABS_ | *4.27±1.25* | *3.69±2.02* | *4.12±1.22* | *1.72±0.81* | *1.78±1.09* | *1.21±1.15* | *5.55±2.00* | *5.84±1.86* | *5.67±1.31* | *2.63±1.45* | *2.81±1.72* | *2.77±0.79* |
| PI_TOTAL_ | *5.71±2.49* | *4.29±1.93* | *4.24±1.52* | *1.37±0.39* | *2.10±0.83* | *1.54±1.29* | *6.40±2.89* | *6.14±2.61* | *6.40±1.87* | *2.56±1.31* | *2.96±1.28* | *2.92±0.95* |

**Supplementary Figure 1.** Graphical analysis of the OJIP chlorophyll fluorescence data measured in maize 4^th^ leaves at Timepoint 2. Plants were well-watered and either treated with 24-*epi*brassinolide (the BR1 variant) or with water (the C variant). The OJIP curve **(A)**, the relative variable fluorescence W_OI_ **(B)** and the difference kinetics ΔW_OJ_ **(C)** and ΔW_OK_ **(D)** are shown. Only the part between the I and P points of the OJIP curve is shown for the W_OI_. ΔW_OJ_ reveals the K-band; ΔW_OK_ reveals the L-band and were calculated from the comparison of BR1 and C variants; the latter are represented by the zero point of the respective y axes in graphs in panels **(C)** and **(D)**. Mean values (n = 8) are shown. a.u. … alternative units.

**
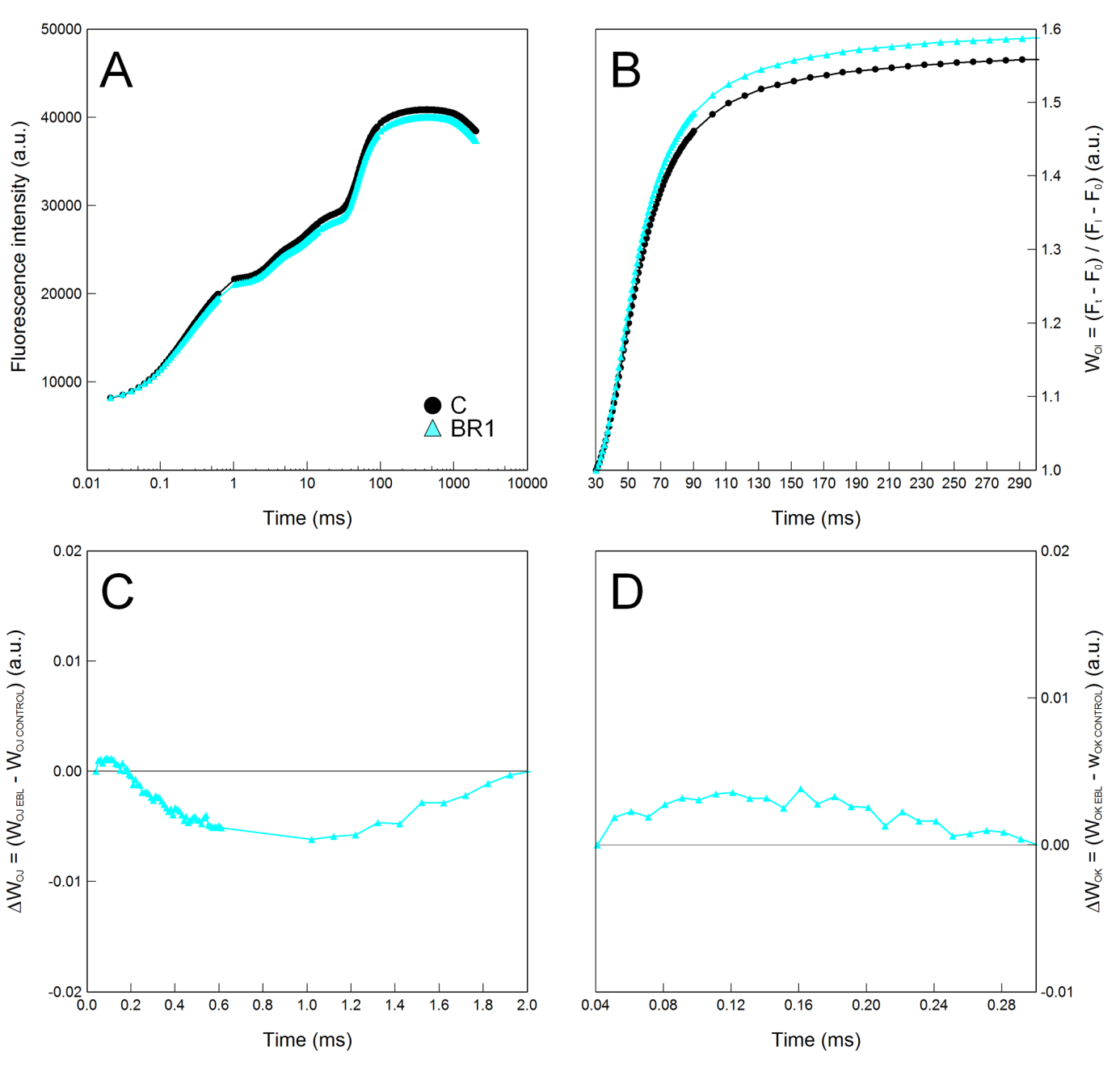
**

**Supplementary Figure 2.** Graphical analysis of the OJIP chlorophyll fluorescence data measured in maize 3^rd^ leaves at Timepoint 3. Plants were either treated with 24-*epi*brassinolide (the BR1 variant) or with water (the C variant) and subjected to normal watering (non-stressed plants) or 7 d of withholding water (stressed plants). The OJIP curve **(A)**, the relative variable fluorescence W_OI_ **(B)** and the difference kinetics ΔW_OJ_ **(C, E)** and ΔW_OK_ **(D, F)** are shown. ΔW_OJ_ and ΔW_OK_ were calculated either from the comparison of the respective BR1 and C variants subjected to the same watering regime **(C, D)** or from the comparison of the respective non-stressed and stressed plants subjected to the same type of treatment **(E, F)**. For other information see legend to Supplementary Figure 1.


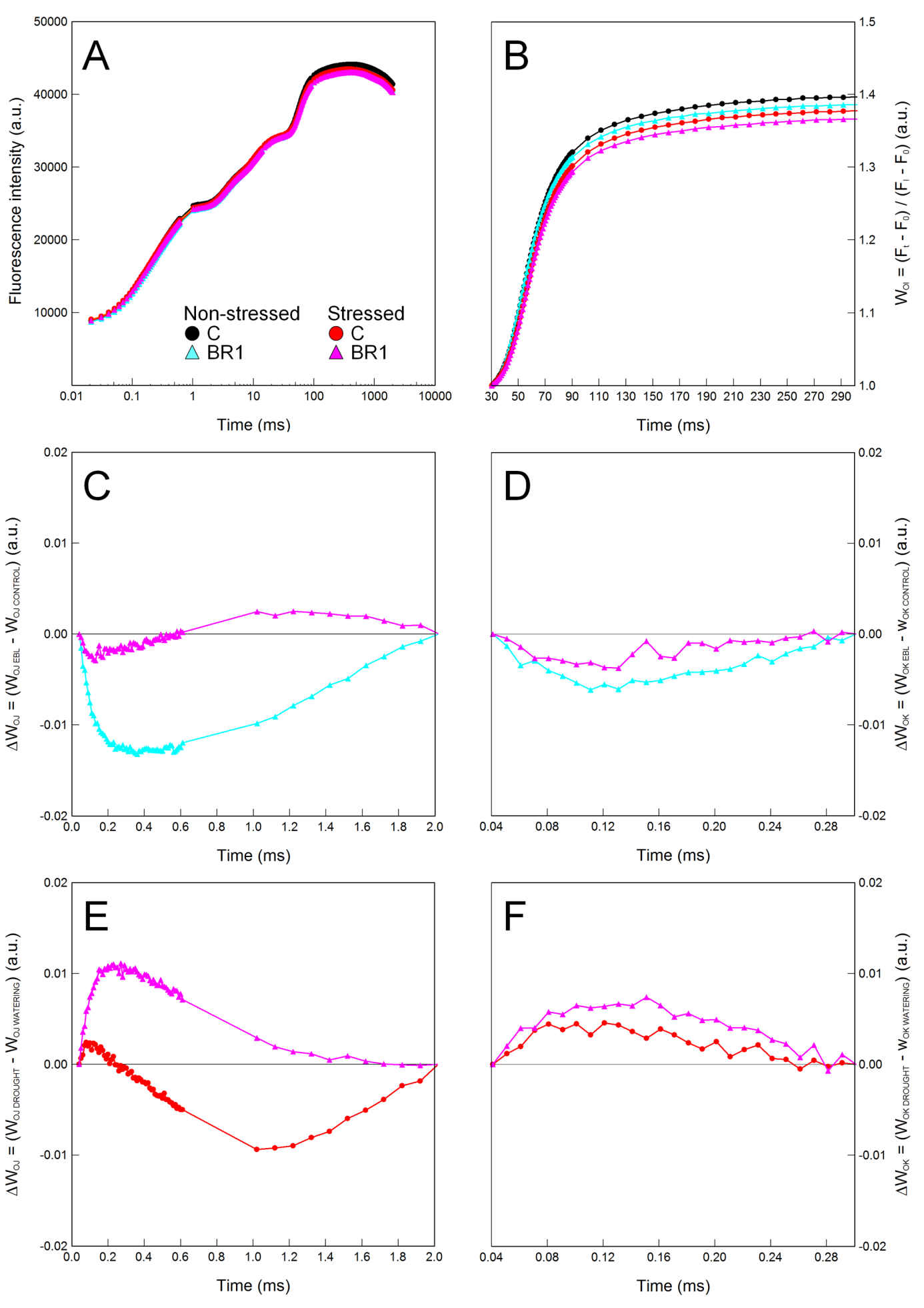

Supplement: Supplementary file 1 [file DataSheet_1.docx]
